# Variability in resistance training trajectories of breast cancer patients undergoing therapy

**DOI:** 10.1007/s00520-024-09001-4

**Published:** 2024-12-10

**Authors:** Maximilian Koeppel, Karen Steindorf, Martina E. Schmidt, Friederike Rosenberger, Joachim Wiskemann

**Affiliations:** 1 Institute of Sports and Sport Science, Heidelberg University, Heidelberg, Germany; 2 Working Group Exercise Oncology, Department of Medical Oncology, National Center for Tumor Diseases Heidelberg (NCT Heidelberg) and Heidelberg University Hospital, a partnership between DKFZ and University Medical Center Heidelberg, Im Neuenheimer Feld 460, 69120 Heidelberg, Germany; 3 Division of Physical Activity, Prevention and Cancer, German Cancer Research Center (DKFZ) and National Center for Tumor Diseases (NCT) Heidelberg, a partnership between DKFZ and University Medical Center Heidelberg, Im Neuenheimer Feld 581, 69120 Heidelberg, Germany

**Keywords:** Exercise oncology, Adjuvant tumor treatment, Response variability, Hierarchical model, Bayesian statistics

## Abstract

**Purpose:**

In resistance training (RT), the change in volume-load from training sessions (TS) to TS is an indicator of training progress. Resulting growth trajectories are likely to differ between individuals. Understanding this variation is important for exercise planning in general, but even more for clinical populations. We investigated this variation in breast cancer patients undergoing treatment.

**Methods:**

Data of 69 patients from two randomized controlled trails were investigated. They conducted a 12-week RT program. We fitted a quadratic Bayesian regression model to the baseline standardized volume-load over the course of the intervention. We allowed all parameters to vary both between exercises and between individuals.

**Results:**

We observed a positive linear component of 0.093 (95% uncertainty interval (UI) 0.058 to 0.120) and a negative quadratic component of − 0.002 (95% UI -0.008 to 0.001) for the mean trajectory of the change in volume-load. For the different exercises, we observed a dispersion for both the linear (0.043, 95% UI 0.018 to 0.082) and the quadratic component (0.002, 95% UI < 0.001 to 0.004). Variation between individual appears to be approximately four times larger. We also observed between-exercise variation within individuals. Extrapolation of the regression model indicates training progression stagnates after 20.6 TS (95% UI 14.8 to 44.4).

**Conclusion:**

There is substantial variation in RT response between breast cancer patients undergoing tumor therapy and in-between exercises. The non-linear trajectory indicates that training progression will eventually plateau, demanding periodization and timely modification.

**Trial registration:**

BEATE Study: NCT01106820, Date: April 20, 2010; BEST Study: NCT01468766, Date: November 9, 2011.

**Supplementary Information:**

The online version contains supplementary material available at 10.1007/s00520-024-09001-4.

## Introduction

Based on a large body of evidence, resistance training (RT) provides several positive effects for cancer patients. However, RT effects do not follow a simple causal stimulus–response relationship between mechanical input and physiological adaptation, but are subject to a complex network of effect modificators [16, 41]. Thus, RT effects may vary between individuals and leads to the classification of individuals into distinct response-categories [1, 3, 10, 12, 17, 25]. In contrast to medical oncology, in which response refers to the efficacy of tumor treatment in reducing tumor size or severity [27] in RT research, the term is used more ambiguously and could refer to several RT-related outcomes of interest, such as the one repetition maximum (1RM), the cross sectional area of a particular muscle [17], or the performance in a functional test [10].

This observation has stimulated scientific endeavors over the last few years and gave rise to controversial discussions [30]. One major criticism is that studies are often confined to comparing data of only two distinct time points with each other where the first point is timed before the start of the exercise intervention and the second one right after the end of the training period. Thus, the two measurements are prone to within-individual variation, such as different mood, motivation, and pain tolerance, but also measurement error which can lead to misestimations [2, 16]. Another statistical artifact would be that extreme values in the first assessment tend to be drawn closer to the mean in the second assessment, resulting in an overestimation of the effect variability [4]. From a more practical perspective, it is well known that responses to RT follow a non-linear time trajectory, with the steepest incline at the beginning of the intervention, which eventually approaches a plateau during the course of the intervention [28]. By ignoring the qualitative differences, linear models tend to underestimate the strength gains in the early stages of training and overestimate the later ones. Furthermore, strength gains do not appear systemic but locally, involving primarily those muscles engaged in the particular exercise [26]. Therefore, it is surprising that the RT response variability within individuals has not received much attention so far.

Although response variability to exercise has been mentioned in several well placed publications, in exercise oncology [11, 18, 37], it did not attract the researchers’ focus of interest. However, the investigation of exercise response variability is especially important for clinical populations, to ensure the optimal care of these populations. Ignoring the effect variability could lead to false expectations for the individual patient, due to an overconfidence in a one-size-fits-all approach [21]. While discussing RT response variability, focusing solely on strength gains limits the assessment of RT efficacy to only one determinant of muscular work. However, individual differences in the number of repetitions performed at a given load can be an important variable in distinguishing training success among individuals.

The purpose of this study is to investigate how the change in volume-load varies between exercises and between individuals undergoing adjuvant cancer treatment over the course of an RT intervention. In addition, we are interested if, and at what point in time, these training-trajectories might reach a plateau.

Due to the nested structure of the data, we fitted a hierarchical Bayesian regression model, which allowed us to estimate the variability between exercises and between individuals. In order to model a potentially non-linear time-volume relationship and estimate the number of TS leading to a plateau, we included a quadratic term to the model.

## Method

### Design and participants

We conducted a secondary analysis by pooling the data of the BEATE (NCT01106820) and the BEST trial (NCT01468766), two randomized clinical trials conducted with physically inactive (< 1 h/week exercising), non-metastatic breast cancer patients (age > 18 years) undergoing adjuvant chemotherapy (BEATE trial) or adjuvant radiation therapy (BEST trial). Detailed information about the study design is described elsewhere [31, 35]. Briefly summarized, both trials investigated the effect of a 12-week machine-based RT intervention on cancer-related fatigue. These studies were approved by the ethics committee (S-012/2009, S-447/2010) and were conducted in accordance with the Declaration of Helsinki, and written informed consent was obtained from all patients.

**Fig. 1:**
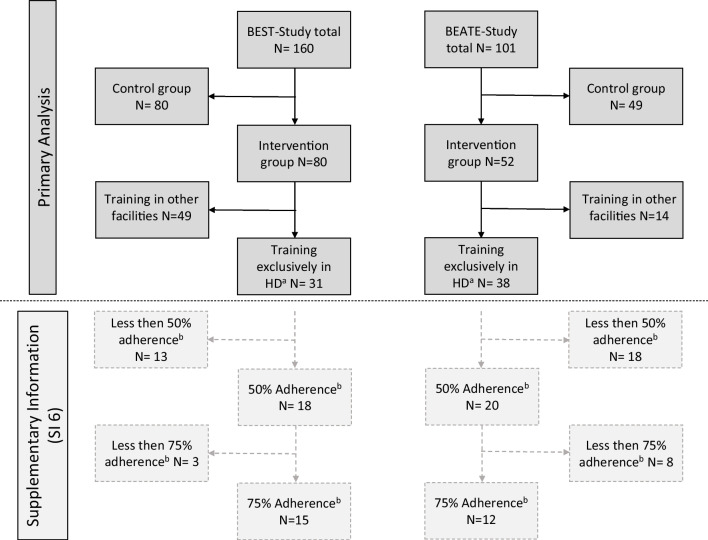
Patient flow. Caption: ^a^HD: Heidelberg, ^b^100% refers to the maximum of 24 training sessions)

A total of 261 patients (101 BEATE, 160 BEST) were enrolled in both trials with 132 assigned to the intervention group (52 BEATE, 80 BEST). While many patients trained at the university’s training facility (41 BEATE, 31 BEST), the patients were also given the option to train in facilities near their homes in order to reduce the barriers for participation. For this analysis, we only included the 69 patients who trained at the study center’s facility to reduce additional sources of variance (Fig. 1). Baseline patient characteristics are outlined in Table 1.

**Table 1:** Patient characteristics

		**Total**		**BEATE**		**BEST**	
TOTAL n		69	(100)	38	(100)	31	(100)
Age (yr)		53.6	(10.5)	52.6	(10.2)	54.9	(10.8)
Days since surgery		57.2	(37.2)	53.6	(17.5)	61.6	(52.2)
BMI (kg*m^−2^)^a^		25.4	(4.7)	24.4	(4.0)	26.7	(5.1)
BMI (kg*m^−2^)^a^	< 25	32	(46.4)	22	(57.9)	10	(32.3)
	25– < 30	25	(36.2)	12	(31.6)	13	(41.9)
	30 +	12	(17.4)	4	(10.5)	8	(25.8)
Smoking Status	Non-smoker^b^	52	(75.4)	27	(71.1)	25	(80.6)
	Quit smoking within past year	11	(15.9)	8	(21.1)	3	(9.7)
	Still smoking at T0	6	(8.7)	3	(7.9)	3	(9.7)
Physical fatigue^c^		41.3	(27.1)	38.5	(23.1)	44.9	(31.3)
Affective fatigue^c^		32.2	(24.3)	26.8	(22.0)	38.9	(25.8)
Cognitive fatigue^c^		33.2	(25.4)	28.4	(23.9)	39.1	(26.3)
Total fatigue^c^		37.8	(21.7)	34.2	(18.6)	42.3	(24.6)
Pre-treatment	Adjuvant chemo^d^	2	(2.9)			2	(6.5)
	Neo-adj. chemo^d^	5	(7.2)			5	(16.1)
	None^d^	24	(34.8)			24	(77.4)
	None^e^	38	(55.1)	38	(100)		
Herceptin therapy at T0	No	65	(94.2)	35	(92.1)	30	(96.8)
	Yes	4	(5.8)	3	(7.9)	1	(3.2)
Hormone therapy at T0	No	51	(73.9)	38	(100)	13	(41.9)
	Yes	18	(26.1)			18	(58.1)

Values are presented as *n* (%) or mean ± SD

^a^Calculated as the weight in kilograms divided by the square of the height in meters

^b^Smoking cessation for at least 1 year

^c^Assessed via Multidimensional Fatigue inventory (MFI-20)

^d^Treatment before enrollment in BEST study

^e^Treatment before enrollment in BEATE study

### Intervention

In accordance with the ACSM exercise guidelines for cancer survivors, currently during the conduction of the study [36], the training was planned to take place twice a week over the course of 12 weeks, resulting in a maximum number of 24 TS. In both studies, the same core exercises were conducted: leg press, leg extension, leg curl, shoulder internal and external rotation, seated row, latissimus pull down, and butterfly. Patients who participated in the BEATE trial also performed butterfly reverse each TS and anteversion or retroversion of the shoulder every other TS. Each exercise was conducted for 1 to 3 sets with 12 repetitions at 60–80% of their hypothetical one repetition maximum, estimated with the Brzycki formula [7]. The training schedule followed a progressive approach, in which the applied load was increased by at least 5% if the prescribed load was successfully lifted for 3 sets of 12 repetitions in three consecutive TS. The TS were supervised by experienced exercise or physical therapists. For more details, please refer to the design [31, 35] and primary endpoint papers [34, 38] as well as the CERT-Checklist (Supplementary Information SI 9).

### Construction of outcome and predictor variables

The goal of the analysis was to investigate the change in volume-load over time. Therefore, the number of the TS was chosen as predictor variable. We excluded the first two TS from each patient since those were considered familiarization sessions. As dependent variable, we chose the volume-load which is constituted by the load, the number of sets, and the number of repetitions per set [29]. Changes in either one of the constituents is an indicator of training progression and is therefore our most comprehensive proxy in determining training progression.

The volume-load per TS for each exercise was z-standardized to the baseline mean and baseline standard deviation of the particular exercise and served as outcome variable for all further analyses. Thus, the resulting regression coefficients can be interpreted and will be referred to as standardized mean differences (SMD). TS were considered valid if more than eight repetitions and more than two sets were performed. If the number of repetitions exceeded 12, volume-load was equalized with the volume-load of the next valid session to avoid misestimations. Management of outliers is discussed in (Supplementary Information SI 1).

### Statistical analysis

We applied a quadratic hierarchical Bayesian regression model with the z-standardized volume-load as outcome variable and number of TS as predictor variable. We added a quadratic term to the model, assuming that training progression does not follow a linear path but will display a steep incline in the first training sessions and eventually approaching a plateau [28]. The model consists of three levels: the individual TS (level 1) which are assigned to the different exercises (level 2), which are then assigned to the patients (level 3). This model allowed all parameters (i.e., intercept, linear component, and quadratic component) to vary on the 2nd and 3rd level and enabled us to investigate the variability of the model parameters between exercises (level 2), between individuals (level 3) but also the between exercises within individuals. Regarding the variability of the RT response trajectories, we focus on the linear component; i.e., a positive linear component describes a positive response, and a negative linear component describes an adverse response.

We chose a Bayesian framework since it allowed us to integrate a large number of already existing information into the prior distribution. Precisely, we used the data of an unpublished meta-analysis of 25 resistance training studies with breast cancer patients and survivors, including a total of 112 effect sizes (Supplementary Information SI 3). Furthermore, the Bayesian analysis does not result in a point estimate for each parameter but in a probability distribution, which allowed us probabilistic interpretations of the parameter estimates [42]. To summarize the posterior distribution of a parameter, we chose the posterior distribution’s mean as the most probable location of the parameter and its 95% uncertainty interval (95% UI), thus, describing the range of the parameter values where the true value can be expected with a 95% probability. For simplicity’s sake, we will be referring to the mean of the posterior distribution as the parameter estimate if not otherwise specified. The analysis was conducted in R with the brms package [8].

### 95% heterogeneity intervals (95% HI)

In accordance with Bolger et al. [6], we also calculated the 95% HI for the exercises (${95\%HI}_{Exercise}$) and the full model (${95\%HI}_{Full}$). The ${95\%HI}_{Exercise}$ states (under the assumption of normality) that the parameter estimate of any exercise for this specific population is expected to lie within this interval with a probability of 95%. For clarification, this differs from the definition of the 95% UI, since the 95% UI describes where the parameter estimate of one exercise can be expected with a 95% probability. Analogously, ${95\%HI}_{Full}$ refers to the range of values where any parameter estimate, regardless of person or exercise, would be expected with a probability of 95%.

### Training sessions until plateau (TS_Plateau_)

The first derivative of the quadratic regression formula allows to calculate the vertex of the function:

$${TS}_{Plateau}=\frac{LinearComponent}{2*QuadraticComponent}$$

This vertex serves as the indicator, where the trajectory will reach its plateau. To model this vertex, 10,000 values were randomly drawn from the posterior distributions of the linear and the quadratic component, resulting in 10,000 pairs of estimates. Each of these pairs was entered into the formula introduced above. This in turn resulted in a probability distribution consisting of 10,000 values for ${TS}_{Plateau}$. In case of profoundly skewed posterior distributions, we chose to report the median of the posterior distribution.

### Model selection and sensitivity analysis

We fitted linear two-level models to the data for each exercise and compared them to their quadratic counterparts using leave-one-out cross-validation. This comparison showed the superiority of the quadratic model (Supplementary Information SI 5). This finding is further supported by examining the frequency of volume-load changes within the first and second halves of the intervention. While we observed a similar proportion of progressions in both halves, weight reductions were substantially more frequent in the second half. These reductions were necessary to adjust for too early or too large increases in training load. Hence, only the results from the quadratic model will be reported. To ensure the transparency and reliability of the analysis, we followed the WAMBS-Checklist [12] (Supplementary Information SI 4 for the analysis with diffuse priors). Finally, we reran the three-level model with only those individuals attending at least 50% of TS (*n* = 38, 53%) and the ones attending at least 75% of TS (Fig. 1) to check if the parameter estimates are skewed by systematically low attendance (Supplementary Information SI 6).

## Results

The 69 analyzed breast cancer patients were 53.6 years old and breast cancer surgery had taken place 57.2 days ago (Table 1). Thirty-eight patients were currently treated with adjuvant chemotherapy and 31 with adjuvant radiotherapy (7 of those patients had received chemotherapy before).

### Main analysis

The main analysis yielded an average increase in the volume-load over the whole training process of SMD = 0.093 (95% UI = 0.058 to 0.120; linear component) per training session. However, every TS needs to be corrected by and average of SMD =  − 0.002 (95% UI = − 0.008 to − 0.001 (quadric component) per TS^2^) (Table 2). The raw baseline volume-load for all exercises is provided in the Supplementary Information SI 10.

**Table 2:** Summary statistics main analysis

	**Mean**	**SD**	**-95% UI**	** + 95% UI**
**Population level effects**				
Intercept	0.049	0.048	− 0.050	0.142
Linear component	0.093	0.016	0.058	0.120
Quadratic component	− 0.002	< 0.001	− 0.003	> − 0.001
**Variation between exercises (as standard deviation)**				
Intercept	0.047	0.038	0.002	0.142
Linear component	0.043	0.016	0.018	0.082
Quadratic component	0.002	0.001	< 0.001	0.004
**Variation between individuals (as standard deviation)**				
Intercept	1.007	0.030	0.951	1.067
Linear component	0.155	0.007	0.142	0.168
Quadratic component	0.008	< 0.001	0.007	0.009

### Variability between exercises

**Fig. 2:**
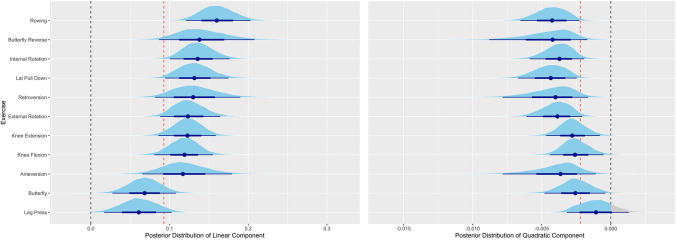
Posterior distribution of **a** the linear and **b** quadratic component for each exercise. Caption: The dashed black line marks the zero line, and the dashed red line the population level estimate

Between exercises, we observed considerable variability regarding the progression of volume-load over time. The standard deviations are SD= 0.043 (95% UI = 0.018 to 0.082) for the linear component and 0.002 (95% UI = 0.001 to 0.004) for the quadratic component. Notably, the posterior distributions of the linear components for all exercises are to the right of the zero line, indicating an average positive training response across exercises (Fig. 2 and Table 3). This is also supported by the 95%HI_Exercise_ analysis showing a range from 0.013 SMD (95% UI = − 0.094 to 0.077) to 0.175 SMD (95% UI = 0.140 to 0.234) and a median coefficient of variation of 45% (95% UI = 19 to 88%) for volume-load increase over time (quadratic component ranges from − 0.006 SMD (95% UI =  − 0.009 to − 0.003) to 0.001 SMD (95% UI =  − 0.002 to 0.007)). The variation between exercises also has an impact on the proportion of individuals who display a positive response to a particular exercise; i.e., the proportion of individuals whose posterior distribution’s median is positive, ranging from 77% for the rowing exercise to 98% for the butterfly exercise (Supplementary Information SI 7 & 8).

**Table 3:** Summary statistics of the posterior distribution of the linear and quadratic component for each exercise

	**Linear component**				**Quadratic component**			
**Exercise**	**Mean**	**SD**	**95% UI**	**95% UI**	**Mean**	**SD**	**95% UI**	**95% UI**
Anteversion	0.119	0.029	0.065	0.179	− 0.004	0.002	− 0.008	− 0.001
Butterfly	0.068	0.021	0.028	0.108	− 0.003	0.001	− 0.005	− 0.000
Butterfly reverse	0.141	0.031	0.087	0.208	− 0.005	0.002	− 0.009	− 0.002
External rotation	0.124	0.019	0.088	0.164	− 0.004	0.001	− 0.006	− 0.002
Internal rotation	0.136	0.019	0.100	0.176	− 0.004	0.001	− 0.006	− 0.002
Knee extension	0.119	0.019	0.081	0.155	− 0.003	0.001	− 0.005	− 0.001
Knee flexion	0.123	0.019	0.086	0.159	− 0.003	0.001	− 0.004	− 0.001
Latissimus pull	0.132	0.021	0.094	0.175	− 0.004	0.001	− 0.007	− 0.002
Leg press	0.061	0.022	0.017	0.103	− 0.001	0.001	− 0.003	0.001
Retroversion	0.131	0.028	0.081	0.190	− 0.004	0.002	− 0.008	− 0.002
Rowing	0.161	0.021	0.121	0.203	− 0.004	0.001	− 0.007	− 0.002

### Variability between individuals

Volume-load variability between individuals over all exercises was SD = 0.155 (95% UI = 0.142 to 0.168) for the linear component and SD = 0.008 (95% UI = 0.007 to 0.009) for the quadratic component.

The variability component of the volume-load between individuals appears to be 3.6 (95% UI = 3.3 to 3.9, linear component) and 4.0 (95% UI = 3.5 to 4.5; quadratic component) times larger than the size of the variation between exercises. Considering the total variation, i.e., individual variation across exercises, SMD ranges from − 0.290 SMD (95% UI = − 0.399 to − 0.224) to 0.478 SMD (95% UI = 0.437 to 0.541) for the linear component and between − 0.021 SMD (95% UI = − 0.025 to − 0.018) to 0.016 SMD (95% UI = 0.013 to 0.022) for the quadratic component indicating a higher impact of variability between individuals vs. different exercises.

### Variability within individuals

**Fig. 3:**
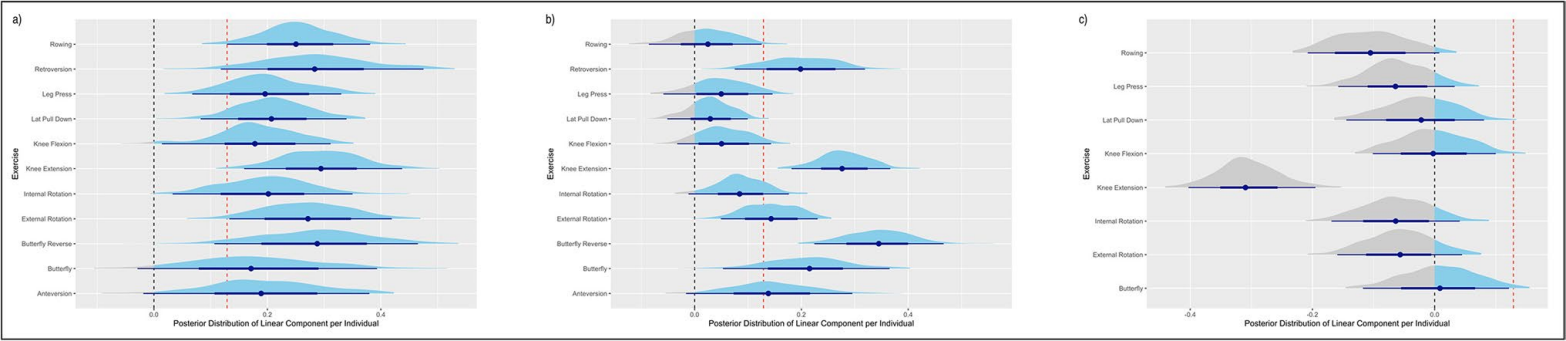
Variation of posterior distributions per exercise within individuals. Exemplary plots for 3 patients: **a** Above average response, **b** average response, **c** below average response. *Caption:* The dashed black line marks the zero line, and the dashed red line the population level estimate. A blue area under the curve characterizes positive parameter values, whereas a grey area under the curve characterizes negative values

The median variability for the volume-load within individuals (variability of volume-load development over time between different exercises within an individual) was 0.121 SMD and ranged from 0.066 to 0.277 (see Fig. 3). This variation was positively correlated with the magnitude of the individuals’ mean progression in volume-load (*r* = 0.39, 95% confidence interval 0.17 to 0.58). Only 13 individuals (19%) showed a negative volume-load development in more than half of the exercises. Of those patients, only one showed a negative volume-load development for all eight exercises and another individual showed seven out of eight. Twenty-one (31%) displayed a positive volume-load development for all exercises and 17 (25%) only a negative volume-load development in one out of eight excises.

### Training sessions until plateau

**Fig. 4:**
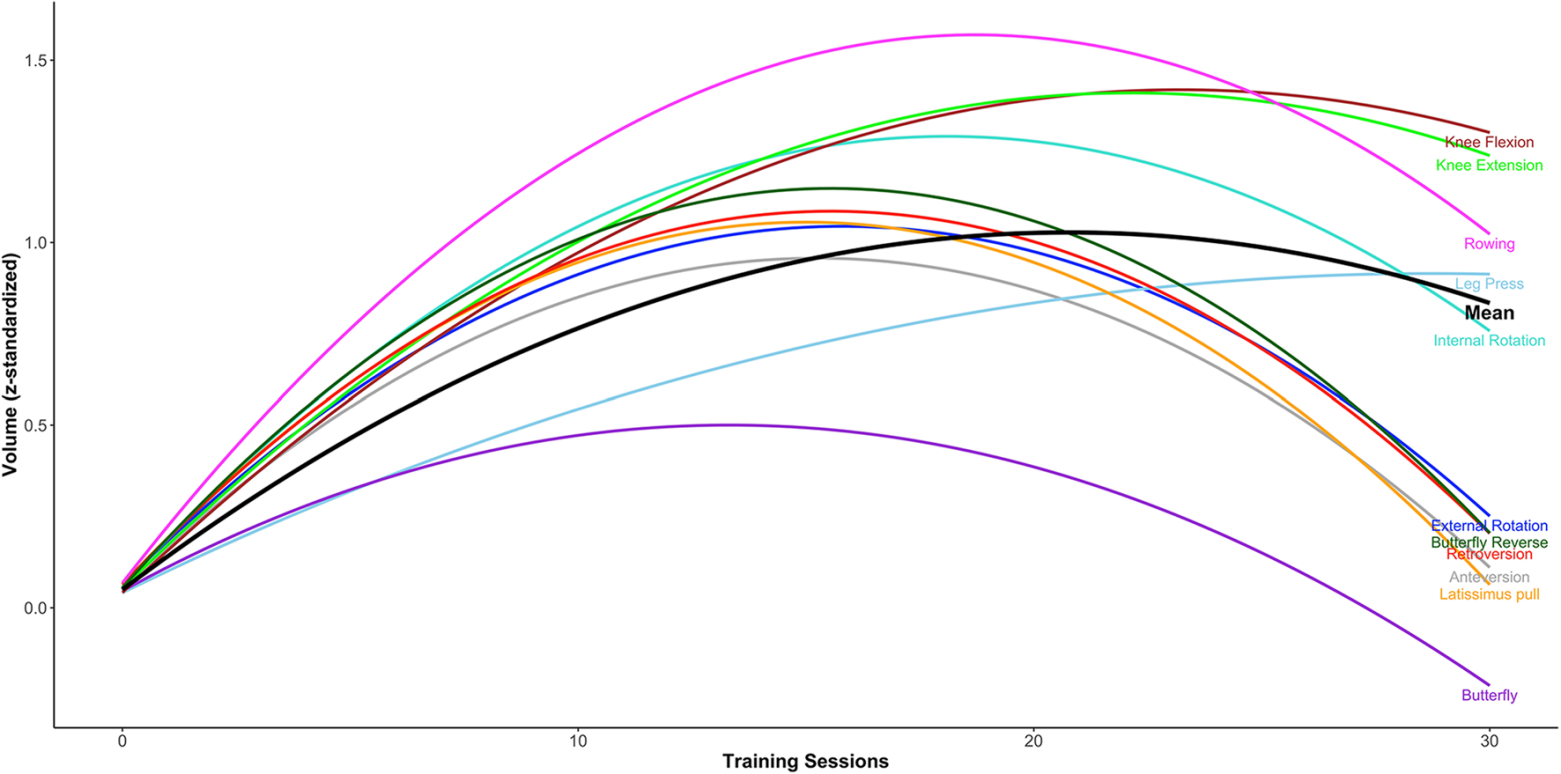
Predicted quadratic trajectory for each exercise. Caption: Due to the quadratic model the volume-load will decrease after it reached the peak. This is certainly not a depiction of the real training trajectory

Independent of a particular exercise, the number of training session needed to reach a volume-load plateau was 20.6 TS (95% UI = 14.8 to 44.4). Across exercises, the butterfly exercise reaches the plateau earliest, with a median time of 13.4 TS (95% UI = : 5.7 to 47.6). The knee extension exercise reaches the plateau latest, with a median of 22.9 TS (95% UI = : 13.1 to 68.9). (Fig. 4). As the 95% UI of the different exercises show a large overlap, no conclusive statements can be made as to whether the time until the plateau is reached differs substantially between the exercises.

## Discussion

Within the last two decades, RT has emerged as a valuable support measure for cancer patients in fighting tumor and therapy-associated side effects [9]. However, these benefits are subject to substantial variation. To the best of our knowledge, this is the first study that systematically investigates RT response variability in breast cancer patients undergoing cancer therapy. We fitted a three-level hierarchical model to the data and estimated separate regression models for each exercise and individual, besides the average trend. We chose the baseline standardized volume-load as dependent variable, which is constituted by the load, the number of sets, and the number of repetitions per set, since changes in either one of the constituents are an indicator of training progression. As expected, we observed positive average responses for all exercises. Additionally, the analysis revealed variability between exercises with a coefficient of variation of about 45% with respect to the average effect. This variation is also evident in the different proportion of individuals responding to the particular exercises. For instance, the butterfly exercise displayed a positive response in all but one participant (98%) whereas roughly a quarter of participants (23%) showed no increase in the rowing exercise (Table 4). With regard to the individual responses, only one case showed a negative time trajectory across all exercises. Thirteen more cases (19%) showed a decline in at least half of the exercises, whereas more than half of participants (55%) displayed no more than one negative trend across exercises. Still, the high proportion of adverse responses appears surprising at first; however, it is crucial to acknowledge that this analysis was conducted in cancer patients undergoing adjuvant treatment. Tumors and their treatment inhibit anabolic pathways while enhancing catabolic pathways in the muscle cell [23]. In a recent systematic review of randomized controlled trials, we investigated the change in body composition in cancer patients undergoing exercise therapy [20]. In a subgroup analysis, we observed a pooled loss in lean body mass in the non-exercising control group, which is in line with other observations [32]. This indicates that, in contrast to the general population where one would expect a maintenance in fitness over a relatively short period of time, in cancer patients, a decline in muscle mass [32] and function has to be expected if not actively counteracted via exercise [13, 19]. From this perspective, the high number of positive responses indicates that most patients did not only prevent the expected functional and structural decline but overcame the negative trend. Thus, instead of dichotomizing participants in responders and non-responders, we suggest to trichotomize the response continuum in clinical populations that are at risk of losing muscle quality: first, those who are insensitive to the stimulus and align with what would be expected in the control condition; second, those who are successful in preventing the decline in strength and muscle mass; and third, those who respond to the stimulus by advancing beyond baseline. Therefore, in a population at risk of accelerated loss of muscle mass and strength, avoiding any decline of the magnitude of the counterfactual control appears to be an accomplishment.

**Table 4:** Extrapolated posterior distribution for time until plateau for each exercise

	**Posterior mean**	**Posterior median**	**Posterior SD**	**95% UI Neg**	**95% UI Pos**
Anteversion	17.8	15.9	8.5	7.3	40.4
Butterfly	16.3	13.4	11.0	5.7	47.6
Butterfly reverse	17.6	16.3	7.1	7.9	35.1
External rotation	16.7	16.0	4.6	9.8	27.5
Internal rotation	19.2	18.3	5.2	11.4	31.7
Knee extension	24.8	22.0	10.5	13.0	54.3
Knee flexion	27.0	22.9	14.2	13.1	68.9
Latissimus pull	15.8	15.2	4.0	9.6	25.2
Leg press^**a**^	718.5	19.1	106,883.0	-242.3	282.3
Retroversion	17.3	16.1	6.5	8.3	33.5
Rowing	19.7	18.9	5.0	12.3	31.5

^a^The extreme parameter estimates observed for leg press can be attributed to the small proportion of values very close to zero for the quadratic term

As hypothesized, the analysis yielded a positive linear and a negative quadratic component for all exercises, resulting in important practical implications. First, the model revealed that all exercises reach a plateau after about 20 TS or 10 weeks with two weekly TS. The majority of studies in exercise oncology and their corresponding guidelines [9, 36] neglect to periodize exercise regimes, despite recommendations for such practices in healthy and athletic populations [33]. This oversight might explain why several systematic reviews have failed to identify a positive relationship between study duration and increases in muscle function or strength beyond 12 weeks of intervention [22, 24, 39]. Notably, the incorporation of periodization regimens is recommended not only for healthy and athletic populations but also in the exercise oncology literature [15].

Second, conventional progression approaches, such as increasing the load by a fixed weight, i.e., a linear progression model or increasing the load by a fixed proportion of the training load (e.g., 2–10%) [33], i.e., an exponential progression model, mismatch the empirical volume-time trajectory. This incongruence might lead to a non-optimal load of the muscles. Based on our analysis, the magnitude of the overload should be reduced with each ongoing progression, by a small increment of approximately 4% of the initial progression of roughly 0.1 SMD which in our data equals roughly 3–5% of the initial load.

For example, if an initial load of 50 kg was selected and a conventional load increase of 5% was chosen, the training weight after 5 progressions would be 64 kg with this classic progression scheme. However, if the correction factor of 4% per progression is introduced, the training weight after 5 progressions is only 61.5 kg. Regarding the considerable adjustments, i.e., the reduction in training load that was observed in the second half of the intervention after increasing the load, compared to the first half, such a correction factor seems not only reasonable but may also facilitate the overall progress of the patients. As an alternative to the quadratic formula provided in the result section, we also constructed a modified exponential growth model displayed in the Supplementary Information SI 10. Future studies should investigate the efficacy of periodization and progressive overload schemas via confirmatory study designs in cancer patients.

There are several limitations to our study. First, it is extremely difficult to operationalize response to resistance exercises in a comprehensive manner [14, 40, 41]. Volume-load as outcome variable has the advantage that it incorporates several progression indicators. However, all three indicators are not independent and a change in one will automatically be reflected in the others. Second, we chose a quadratic function because of the curvilinear behavior of training adaptations. This allowed us to calculate the vertex of the function and to estimate when the training trajectory reaches its plateau. Nevertheless, the quadratic function, since it drops after the vertex, does not mimic the real training trajectory. More precise trajectories could be modeled by applying a proper growth function. Third, the impact of potential predictor variables such as age, therapy status, and severity of the disease needs to be investigated in further research to improve the personalization of exercise routines and provide therapists with more realistic expectations about their patients’ improvements.

Ultimately, improvements in patient-centered variables, such as quality of life or cancer-related fatigue, are the primary goal of exercise intervention, whereas increases in training variables are only of subordinate interest. Thus, to optimize training programs for cancer patients, we need a better understanding of the causal relationship between resistance training, strength, and other health outcomes.

## Conclusion

Despite some variation, breast cancer patients undergoing cancer treatment can improve their strength without limitations to specific exercises and independent of the recruited muscles. Still, therapists should be aware of the demonstrated differences in patients’ training progression and the non-linearity of the training progression so that they act as needed. Eventually, a more personalized approach is needed, which requires closer monitoring of patients and a high expertise in therapists with the fundamentals of RT and the utilization of alternative RT methods [5].

## Supplementary Information

Below is the link to the electronic supplementary material.

Supplementary file1 (DOCX 29 KB)

Supplementary file2 (DOCX 19 KB)

Supplementary file3 (DOCX 797 KB)

Supplementary file4 (DOCX 21 KB)

Supplementary file5 (DOCX 28 KB)

Supplementary file6 (DOCX 26 KB)

Supplementary file7 (DOCX 19 KB)

Supplementary file8 (PPTX 3055 KB)

Supplementary file9 (DOCX 12 KB)

Supplementary file10 (DOCX 10 KB)

Supplementary file11 (DOCX 37 KB)

## Data Availability

The datasets generated for this study are available on request to the corresponding author.
