# Supplementary material for: Variability in resistance training trajectories of breast cancer patients undergoing therapy
Source: Support Care Cancer. 2024 Dec 10;33(1):12. doi: 10.1007/s00520-024-09001-4 (PMC11631991; doi:10.1007/s00520-024-09001-4)
Supplement: Supplementary file 1 — Supplementary file1 (DOCX 29 KB) [file 520_2024_9001_MOESM1_ESM.docx]

**Variability in resistance training trajectories of breast cancer patients undergoing therapy**

Maximilian Koeppel^1,2^, Karen Steindorf^3^, Martina E. Schmidt^3^, Friederike Rosenberger^2^, Joachim Wiskemann^2^

^1^Institute of Sports and Sport Science, Heidelberg University, Heidelberg, Germany

^2^Working Group Exercise Oncology, Department of Medical Oncology, National Center for Tumor Diseases Heidelberg (NCT Heidelberg) and Heidelberg University Hospital, Heidelberg Germany

^3^Division of Physical Activity, Prevention and Cancer, German Cancer Research Center (DKFZ) and National Center for Tumor Diseases (NCT) Heidelberg, Heidelberg, Germany

*Supplementary Information 1 – Outlier Analysis*

**Selection Criteria**

- Identification of outliers via function „boxplot.stats“.
- Far Outliers k= 3.0 times the interquartile range outside of the box
- Regular Outliers k= 1.5 times the interquartile range outside of the

**Results**

*Variable: Resistance*

Table 1. Number and Proportion of outlier values

| **Exe** | **LP** | **KE** | **KF** | **Row** | **Lat** | **IR** | **ER** | **AV** | **RV** | **RB** | **BF** | **RM** |
| --- | --- | --- | --- | --- | --- | --- | --- | --- | --- | --- | --- | --- |
| **Total** | **933** | **927** | **929** | **931** | **933** | **917** | **910** | **216** | **2273** | **220** | **718** | **429** |
| **K=3.0** | 0 | 10  (1,1%) | 0 | 0 | 0 | 0 | 0 | 0 | 0 | 0 | 0 | 0 |
| **K=1.5** | 0 | 14^1^  (1,5%) | 15  (1,6%) | 5  (0,5%) | 0 | 0 | 7  0,8% | 5  (2,3%) | 0 | 0 | 0 | 0 |

LP: Leg press, KE: Knee extension, KF: Knee flexion, Row: Rowing, Lat: Latissimus Pull, IR: Internal rotation, ER: External rotation, AV: Anteversion, RV: Retroversion, RB: Reverse Butterfly, BF: Butterfly, RM: Rowing Machine

^1^after correction of the 10 far out values

1. **Inspection of the ten far-out values in knee extension exercise**

- All ten values belong to the same person
- Considering that the 95%-Percentile Range of the KE resistance data reaches from 8.8kg to 35.2kg, it appears to be extremely unlikely that the documented values of 52.8 – 67.5 were measured in kg. Transferring them into lbs, would equal a range of 23.9 – 30.6 lbs, which fits well to the remaining distribution.
- Therefore, the ten outlier values were divided by 2.2 to convert lbs into kg

1. **Inspection of regular outliers**
   - *BEATE-27:*
     1. Patient was responsible for 8 outliers in KF and all outlier values in Row and AV.
     2. In all but one exercise (ER) the average load of BEATE-27 were beyond the 75^th^-Percentile (Table 1).
     3. Therefore we did not suspect the identified outliers are actual anomalies in the data.

Table 2. 75th Percentile values per exercise and mean per exercise of patient BEATE-27

| **Exe** | **LP** | **KE** | **KF** | **Row** | **Lat** | **IR** | **ER** | **AV** | **RV** | **RB** | **BF** | **RM** |
| --- | --- | --- | --- | --- | --- | --- | --- | --- | --- | --- | --- | --- |
| **Med** | **45,0** | **17,6** | **11,0** | **15,0** | **20,0** | **6,0** | **5,0** | **4,0** | **6,0** | **7,0** | **2,2** | **15,0** |
| **75^th^ P.** | **61,0** | **26,4** | **11,0** | **20,0** | **25,0** | **7,0** | **6,0** | **5,0** | **7,0** | **8,0** | **6,0** | **NA** |
| **BEATE-27** | 71,4 | 30,5 | 16,5 | 26,3 | 26,8 | 7,3 | 5,0 | 8,5 | 8,8 | 8,7 | 9,5 | NA |

LP: Leg press, KE: Knee extension, KF: Knee flexion, Row: Rowing, Lat: Latissimus Pull, IR: Internal rotation, ER: External rotation, AV: Anteversion, RV: Retroversion, RB: Reverse Butterfly, BF: Butterfly, RM: Rowing Machine

Green: below Median, Yellow: between median and 75^th^ percentile, Blue: above 75^th^ percentile

**Remaining Outliers:**

- All but one (KE) of the remaining outliers (13 in KE, 7 in KF and 7 in ER) could be assigned to three patients (BEATE-4, BEST-14, BEST-5).
  - As it can be seen in Table 2, Patients BEATE-4, BEST-14 and BEST-5 display above average strength.
    1. *BEATE-4 (KE):* Excluding the outlier value, the patient displays an average load above the sample’s 75^th^ percentile for six (60%) out of 10 exercises and in the three (27%) of the remaining 4 exercises values above the sample’s median. Only the mean for RB is below, but basically identical with the median of the sample.
    2. *BEST-14 (KF):* Excluding the outlier value, the patient displays an average load above the sample’s 75th percentile in one exercise (14%). In the remaining six exercises (86%) the average load is above the sample’s median.
    3. *BEST-5 (ER):* Excluding the outlier value, the Patient displays an average load above the sample’s 75th percentile in two exercises (29%). In four (57%) of the remaining seven exercises the average load is above the sample’s median. Furthermore, BEST-5 also has an extraordinary high mean value for internal rotation which could be correlated to ER.

Table 3. Resistance mean values of outlier producing patients for each exercise

| **Exe** | **LP** | **KE** | **KF** | **Row** | **Lat** | **IR** | **ER** | **AV** | **RV** | **RB** | **BF** |
| --- | --- | --- | --- | --- | --- | --- | --- | --- | --- | --- | --- |
| **Median** | **45,0** | **17,6** | **11,0** | **15** | **20** | **6,0** | **5,0** | **4,0** | **6,0** | **7,0** | **2,2** |
| **75^th^ Per.** | **61,0** | **26,4** | **11,0** | **20,0** | **25,0** | **7,0** | **6,0** | **5,0** | **7,0** | **8,0** | **6,0** |
| **BEATE-4** | 61,0 | 38,9 | 14,6 | 18,9 | 24,3 | 6,9 | 6,4 | 5,5 | 7,4 | 6,9 | 7,0 |
| **BEST-14** | 56,7 | 20,2 | 14,7 | 21,5 | 21,5 | 6,1 | 4,1 | NA | NA | NA | 2,5 |
| **BEST-5** | 45,5 | 17,6 | 5,9 | 22,3 | 21 | 8,6 | 8,1 | NA | NA | NA | 1 |

LP: Leg press, KE: Knee extension, KF: Knee flexion, Row: Rowing, Lat: Latissimus Pull, IR: Internal rotation, ER: External rotation, AV: Anteversion, RV: Retroversion, RB: Reverse Butterfly, BF: Butterfly, RM: Rowing Machine; Median: Median of all Patients, 75th Per.: 75th Percentile of all Patients

Red: Outlier exercise, Green: below Median, Yellow: between median and 75^th^ percentile, Blue: above 75^th^ percentile

- - *BEATE-21 (KE):* The remaining outlier belongs to Patient BEATE-21. As it can be seen from the progression of this patient’s training load in KE (Table 4) the outlier value of 39.6 fits well to the other values. The change from T14 to T15 equals an increase in load of 4.4 kg (12.5%) which seams reasonable. The load increase and the decrease afterwards could be well explained by the the attempt to increase the resistance which lead to the patient’s discomfort.

Table 3. Training Load in kg per training session (TS).

| Data: | T_0_= 21.9; T_1_=26.4; T_2_=26.4; T_3_= 30.8; T_4_= 35.2; T_5_= 35.2; T_6_= 35.2; T_7_= 35.2; T_8_= 35.2; T_9_= 35.2;  T_10_= 35.2; T_11_= 35.2; T_12_= 35.2; T_13_= 35.2; T_14_= 35.2; T_15_= 39.6; T_16_= 35.2; T_17_= 35.2 |
| --- | --- |

**Summary:**

| - The ten far out values are probably the resistance in lbs instead of kg and have been converted. - The outliers for KF, Row and AV are all from the same person who displayed strength levels substantially beyond the sample’s median in all but one exercises. - The remaining outliers are not troublesome either, since they fit well to the remaining data and the patients who produced the outliers display above average strength in the majority of exercises |
| --- |
