# Supplementary material for: Variability in resistance training trajectories of breast cancer patients undergoing therapy
Source: Support Care Cancer. 2024 Dec 10;33(1):12. doi: 10.1007/s00520-024-09001-4 (PMC11631991; doi:10.1007/s00520-024-09001-4)
Supplement: Supplementary file 2 — Supplementary file2 (DOCX 19 KB) [file 520_2024_9001_MOESM2_ESM.docx]

**Variability in resistance training trajectories of breast cancer patients undergoing therapy**

Maximilian Koeppel^1,2^, Karen Steindorf^3^, Martina E. Schmidt^3^, Friederike Rosenberger^2^, Joachim Wiskemann^2^

^1^Institute of Sports and Sport Science, Heidelberg University, Heidelberg, Germany

^2^Working Group Exercise Oncology, Department of Medical Oncology, National Center for Tumor Diseases Heidelberg (NCT Heidelberg) and Heidelberg University Hospital, Heidelberg Germany

^3^Division of Physical Activity, Prevention and Cancer, German Cancer Research Center (DKFZ) and National Center for Tumor Diseases (NCT) Heidelberg, Heidelberg, Germany

*Supplementary Information 2 - Statistical model*

Level 1:

$y_{t,e,i}\sim N\left( \mu,\sigma_{\varepsilon} \right)$ [1]

$\mu=\beta_{0,e,i}+\beta_{1,e,i}*TS+\beta_{2,e,i}*{TS}^{2}$ [2]

Level 2:

$\beta_{0,e,i}\sim N(\gamma_{0,0,i},\tau_{0})$ [3]

$\beta_{1,e,i}\sim N(\gamma_{1,0,i},\tau_{1})$ [4]

$\beta_{2,e,i}\sim N(\gamma_{2,0,i},\tau_{2})$ [5]

Level 3:

$\gamma_{0,0,i}\sim N(\delta_{0,0,0},\varphi_{0})$ [6]

$\gamma_{1,0,i}\sim N(\delta_{1,0,0},\varphi_{1})$ [7]

$\gamma_{2,0,i}\sim N(\delta_{2,0,0},\varphi_{2})$ [8]

In Formula [1] the z-standardized training volume $y$ of a particular TS*,* for an exercise *e* of an individual *i* is assumed to follow a normal distribution *N*. This normal distribution is defined with mean $\mu$ and a standard deviation $\sigma_{\varepsilon}$. The mean µ of this distribution constitutes of a regression model [2] with an intercept $\beta_{0,e,i}$ for an exercise e of an individual i, the linear component ${\beta_{1,e,i}}_{1}$ for an exercise e of an individual i times the TS, and the quadratic component $\beta_{1,e,i}$ for an exercise of an individual i times the TS squared. The standard deviation $\sigma_{\varepsilon}$ refers to the residual variation in z-standardized volumes for each exercise of each individual. Respectively, formula [3], [4] and [5] describe that it is assumed that the parameter estimates $\beta_{0,e,i}$, $\beta_{1,e,i}$ and $\beta_{2,e,i}$ originate from a normal distribution, with the average intercept $\gamma_{0,0,i}$*,* linear component $\gamma_{1,0,i}$ and quadratic component $\gamma_{2,0,i}$ of each individual i across the exercises as mean. The standard deviation $\tau_{0,e}$ represents the variation between exercises regarding the intercept, $\tau_{1,e}$regarding the linear component, or in case of $\tau_{2,e}$ in regards to the quadratic component. Finally, in formula [6], [7] and [8] parameters $\gamma_{0,0,i}$, $\gamma_{1,0,i}$ and $\gamma_{2,0,i}$ are assumed to follow a normal distribution around the corresponding average intercept $\delta_{0,0,0}$, linear component $\delta_{1,0,0}$ or quadratic component $\delta_{2,0,0}$, across exercises and individuals. $\varphi_{0,i}$ , $\varphi_{1,i}$ and $\varphi_{2,i}$ stand for the respective variation between individuals with respect to the within individual variation of exercises.
