# Supplementary material for: Variability in resistance training trajectories of breast cancer patients undergoing therapy
Source: Support Care Cancer. 2024 Dec 10;33(1):12. doi: 10.1007/s00520-024-09001-4 (PMC11631991; doi:10.1007/s00520-024-09001-4)
Supplement: Supplementary file 3 — Supplementary file3 (DOCX 797 KB) [file 520_2024_9001_MOESM3_ESM.docx]

**Variability in resistance training trajectories of breast cancer patients undergoing therapy**

Maximilian Koeppel^1,2^, Karen Steindorf^3^, Martina E. Schmidt^3^, Friederike Rosenberger^2^, Joachim Wiskemann^2^

^1^Institute of Sports and Sport Science, Heidelberg University, Heidelberg, Germany

^2^Working Group Exercise Oncology, Department of Medical Oncology, National Center for Tumor Diseases Heidelberg (NCT Heidelberg) and Heidelberg University Hospital, Heidelberg Germany

^3^Division of Physical Activity, Prevention and Cancer, German Cancer Research Center (DKFZ) and National Center for Tumor Diseases (NCT) Heidelberg, Heidelberg, Germany

*Supplementary Information 3 - Prior Specification and Prior Predictive Check*

**Derivation of Priors**

We conducted an unpublished Bayesian meta analysis incorporating a total of 222 effect sizes measuring the effect of resistance training interventions in 50 randomized controlled trials with cancer patients. Half of the RCTs (n= 25) and the effect sizes (n= 112) were solely conducted in breast cancer patients. Within these 25 studies incorporating only breast cancer patients we estimated an average effect (Standardized mean difference SMD) across studies and measurements of SMD= 0.56 (95% uncertainty interval (UI) 0.49 - 0.64) and a variability (standard deviation of the SMD distribution) of σ= 0.40 (95% UI 0.36 - 0.48). In a second analysis we distinguished between therapy status but not cancer site of patients. Patients undergoing chemotherapy (CT) or radio therapy (RT) displayed lower effects then patients not undergoing acute therapy (CT: SMD= 0.36, 95% UI: 0.24 - 0.48; RT: SMD= 0.39, 95% UI: 0.23-0.55). The variability was similar to the one estimated above (CT: σ= 0.35, 95% UI: 0.27, 0.45; RT: σ= 0.30, 95% UI 0,19-0,49). Since the effect estimate of breast cancer patients is slightly larger then the one estimated across cancer sites, we would expect that the effect for breast cancer patients undergoing therapy is as well slightly larger then the one estimated across cancer sites.

Median duration of the interventions was 12 weeks and median frequency was two training sessions per week, and therefore representative for the data discussed in the manuscript. In a sub-group analysis we found that studies that lasted longer then twelve weeks did not show any incremental benefit to those studies lasting between seven and twelve weeks. Which lead us to the following propositions:

| 1. Model: y= β_1_TS + β_2_TS^2^ (Quadratic Model) 2. First Derivation: y’= β_1_TS + β_2_TS^2^ 3. Mean Effect: SMD= 0.40 4. Peak after 24 Weeks: y(24)= 0.40 | ( 1 )  ( 2 )  ( 3 )  ( 4 ) |
| --- | --- |
| Insert ( 4 ) in ( 1 ) and ( 2 )   1. 0.40 = β_1_ * 24 + β_2_ * 576 2. 0 = β_1_ + 2β_2_ * 24   Thus: β_2_ = β_1_/48 | ( 5 ) |
| Substitute β_2_ in ( 1 ) with ( 5 )   1. 0.40 = β_1_*24 + β_1_/48 * 576    1. 0.40 = β_1_* (24 - 12)    2. β_1_ = 0.4/12 = 0.0333 | ( 6 ) |
| Substitute β_1_ in ( 5 ) with ( 6 )   - 1. ß2 = β_1_/48 = 0.0007 |  |

Thus: we set the mean of the prior distribution for β_1_ to 0.0333 and for β_2_ to 0.0007. Regarding the uncertainty in the prior estimate we chose the variation in effect sizes as reference. As it can seen from the outlines above, the variation σ is roughly the same as the effect size estimate, therefore we chose the standard deviation of the prior estimates to be of equal magnitude as the prior’s mean estimate.

Due to the z-standardization of the outcome variable we chose 0 as the mean estimate of the intercept’s prior and 0.5 as its uncertainty.

For the higher order variation parameters we chose weakly informative priors. i.e. priors that are wide enough to give the data maximum weight while limiting the parameter space to a reasonable width. For instance we expected that it is unlikely that the mean estimate of an exercise would be negative thus we centered the prior distribution for the between exercise variation of the linear component to be half the average effect. Analogously for the quadratic component we chose half the average effect.

In case of the variation between individuals we chose to double the variation between exercises. This would result in the conservative assumption that 16% of individuals would display a negative mean response to the training intervention. In both cases we added a large uncertainty of 3-4 times the estimates as the priors standard deviation.

We eventually ended up with the following priors:

*Table S2.1. Final choice of priors*

| **Coefficient** | **Distribution** | **Parameters** |
| --- | --- | --- |
| ***Population Level Effect, Constants*** | | |
| Intercept | Normal | 0, 0.5 |
| Linear Component | Normal | 0.033, 0.033 |
| Quadratic Component | Normal | -0.0007, 0.0007 |
| ***Variation between Exercises, Standard Deviation*** | | |
| Intercept | Cauchy | 0, 1 |
| Linear Component | Cauchy | 0.017, 0.1 |
| Quadratic Component | Cauchy | 0.0004, 0.005 |
| ***Variation between Individuals, Standard Deviation*** | | |
| Intercept | Cauchy | 0, 1 |
| Linear Component | Cauchy | 0.033, 0.1 |
| Quadratic Component | Cauchy | 0.0007, 0.1 |

**Prior Predictive Check (PPC)**

**
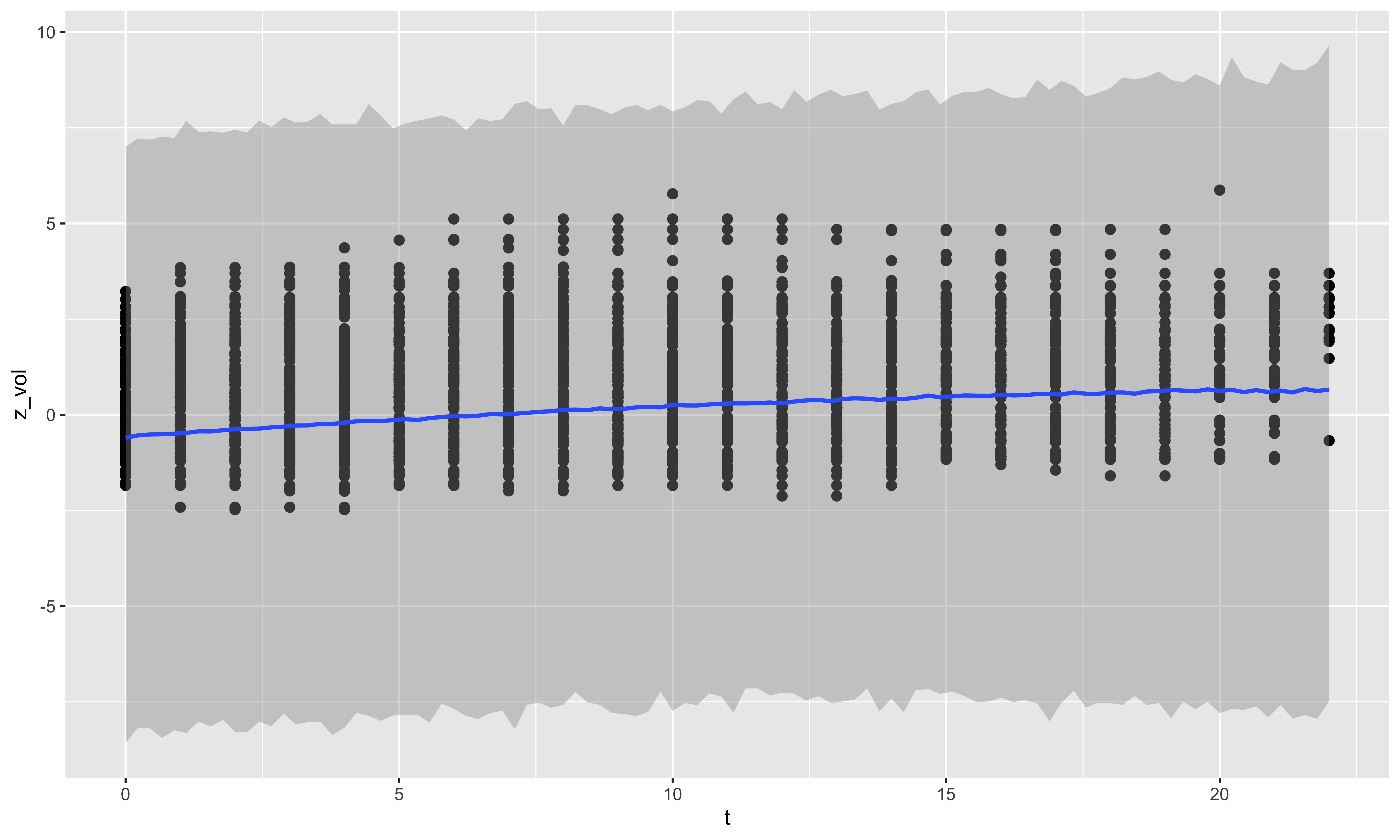
**Due to the hierarchical structure of the data and the high complexity of the model it is difficult to predict the behavior of the model. Therefore it is important to conduct a PPC to investigate if the resulting parameter estimates resulting from modeling the priors alone makes sense (Table S2.2). In the second step, the model is used to predict the range of potential values that are in line with the prior choices and graphically compared with the actual values (Fig. S2_1). As it can be seen in Fig. S2.1 the data predicted from the chosen priors include all observed values.

*Table S2.2. Model resulting from the prior choice*

|  | Posterior Mean | Posterior SD | -95% UI | +95% UI |
| --- | --- | --- | --- | --- |
| **Population Level Effect, Constants** | | | | |
| Intercept | -0,2338 | 0,6068 | -1,4196 | 0,9613 |
| Linear Component | 0,0333 | 0,0329 | 0,0308 | 0,0975 |
| Quadratic Component | -0,0007 | 0,0007 | -0,0021 | 0,0007 |
| **Variation between Exercise, Standard Deviation** | | | | |
| Intercept | 3,3527 | 45,1704 | 0,0172 | 13,0969 |
| Linear Component | 0,2340 | 7,2569 | 0,0023 | 0,6669 |
| Quadratic Component | 0,0031 | 0,0316 | <0,0001 | 0,0136 |
| **Variation between Individuals, Standard Deviation** | | | | |
| Intercept | 2,2824 | 19,3741 | 0,0205 | 11,5644 |
| Linear Component | 0,2580 | 1,7150 | 0,0041 | 1,2312 |
| Quadratic Component | 0,0100 | 0,1605 | 0,0001 | 0,0309 |
