# Supplementary material for: Variability in resistance training trajectories of breast cancer patients undergoing therapy
Source: Support Care Cancer. 2024 Dec 10;33(1):12. doi: 10.1007/s00520-024-09001-4 (PMC11631991; doi:10.1007/s00520-024-09001-4)
Supplement: Supplementary file 4 — Supplementary file4 (DOCX 21 KB) [file 520_2024_9001_MOESM4_ESM.docx]

**Variability in resistance training trajectories of breast cancer patients undergoing therapy**

Maximilian Koeppel^1,2^, Karen Steindorf^3^, Martina E. Schmidt^3^, Friederike Rosenberger^2^, Joachim Wiskemann^2^

^1^Institute of Sports and Sport Science, Heidelberg University, Heidelberg, Germany

^2^Working Group Exercise Oncology, Department of Medical Oncology, National Center for Tumor Diseases Heidelberg (NCT Heidelberg) and Heidelberg University Hospital, Heidelberg Germany

^3^Division of Physical Activity, Prevention and Cancer, German Cancer Research Center (DKFZ) and National Center for Tumor Diseases (NCT) Heidelberg, Heidelberg, Germany

*Supplementary Information 4 – Sensitivity analysis with diffuse priors*

**Background**

To evaluate the impact of the priors we rerun the analysis using the diffuse default priors provided by the

r package brms. Hereby the impact of the prior is minimized.

*Table S3.1 Diffuse Priors for the model parameters*

| Coefficient | Level | Distribution | Parameters |
| --- | --- | --- | --- |
| Intercept | 1 | Flat | |
| t | 1 | Flat | |
| t^2 | 1 | Flat | |
| sd(intercept) | 2 | Student t | 3, 0, 2.5 |
| sd(t) | 2 | Student t | 3, 0, 2.5 |
| sd(t^2 | 2 | Student t | 3, 0, 2.5 |
| sd(intercept) | 3 | Student t | 3, 0, 2.5 |
| sd(t) | 3 | Student t | 3, 0, 2.5 |
| sd(t^2 | 3 | Student t | 3, 0, 2.5 |

**Result**

Compared to the weakly informative and empirical priors used in the main analysis the diffuse priors result in meaningful different results. First, the linear component of the population effect in the main manuscript appears roughly double the size (t= 0.093, 95%UI 0.058, 0.120) compared to the one displayed here (0,051, 95%UI 0.034, 0.070). This is even more so for the variation between exercises. Here the estimate in the main manuscript is three times 2.7 times the size of the one resulting from the diffuse prior analysis. With respect to the population level estimate the analysis utilizing diffuse priors results in a coefficient of variation of 31% compared to the 46% reported in the main analysis.

*Table S3.2 Result of the analysis using diffuse priors*

|  | Posterior Mean | Posterior SD | -95% UI | +95% UI |
| --- | --- | --- | --- | --- |
| **Population Level Effect, Constants** | | | | |
| **Intercept** | 0,017 | 0,048 | -0,076 | 0,111 |
| **Linear Component** | 0,051 | 0,009 | 0,034 | 0,070 |
| **Quadratic Component** | -0,001 | 0,001 | -0,002 | -0,001 |
| **Variation between Exercises, Standard Deviation** | | | | |
| **Intercept** | 0,048 | 0,040 | 0,001 | 0,148 |
| **Linear Component** | 0,016 | 0,009 | 0,001 | 0,039 |
| **Quadratic Component** | 0,001 | <0,001 | 0,002 | 0,113 |
| **Variation between Individuals, Standard Deviation** | | | | |
| **Intercept** | 0,987 | 0,033 | 0,926 | 1,054 |
| **Linear Component** | 0,109 | 0,006 | 0,098 | 0,121 |
| **Quadratic Component** | 0,007 | 0,001 | 0,006 | 0,008 |

**Conclusion**

Although the priors have an influence on the model, we do not consider them as troublesome since the main message of the paper regarding a substantial variation between the exercises and individuals still holds

for the alternative analysis. Furthermore, the quadratic nature of the trajectory has been confirmed in both analysis. We still stick to the analysis presented in the main analysis based on the thorough definition of the prior distribution based on a meaningful amount of data.
