# Supplementary material for: Variability in resistance training trajectories of breast cancer patients undergoing therapy
Source: Support Care Cancer. 2024 Dec 10;33(1):12. doi: 10.1007/s00520-024-09001-4 (PMC11631991; doi:10.1007/s00520-024-09001-4)
Supplement: Supplementary file 5 — Supplementary file5 (DOCX 28 KB) [file 520_2024_9001_MOESM5_ESM.docx]

**Variability in resistance training trajectories of breast cancer patients undergoing therapy**

Maximilian Koeppel^1,2^, Karen Steindorf^3^, Martina E. Schmidt^3^, Friederike Rosenberger^2^, Joachim Wiskemann^2^

^1^Institute of Sports and Sport Science, Heidelberg University, Heidelberg, Germany

^2^Working Group Exercise Oncology, Department of Medical Oncology, National Center for Tumor Diseases Heidelberg (NCT Heidelberg) and Heidelberg University Hospital, Heidelberg Germany

^3^Division of Physical Activity, Prevention and Cancer, German Cancer Research Center (DKFZ) and National Center for Tumor Diseases (NCT) Heidelberg, Heidelberg, Germany

*Supplementary Information 5 - Comparison of quadratic and linear models*

*Table S5.1. Summary statistic and model comparison for the quadratic and linear two level model*

|  | **Quadratic Two Level Model** | | | | | | **Linear Two Level Model** | | | | | **Comparison** | | | |
| --- | --- | --- | --- | --- | --- | --- | --- | --- | --- | --- | --- | --- | --- | --- | --- |
| **Exercise** | **Parameter** | **Estimate** | **Se** | **95%-UI** | | **elpd loo^a^** | **Estimate** | **Se** | **95%-UI** | | **elpd loo^a^** | **elpd Diff.^b^** | **Se Diff.^c^** | **Stand. Diff.^d^** |  |
| **Anteversion** | **Intercept** | 0,046 | 0,163 | -0,275 | 0,368 | -156,25 | 0,076 | 0,185 | -0,326 | 0,425 | -172,15 | -15,9 | 7,62 | -2,09 |  |
|  | **Linear Comp.** | 0,068 | 0,026 | 0,016 | 0,117 |  | 0,022 | 0,008 | 0,006 | 0,037 |  |  |  |  |  |
|  | **Quad. Comp** | -0,001 | 0,001 | -0,002 | 0,001 |  | NA | NA | NA | NA | NA |  |  |  |  |
| **Butterfly** | **Intercept** | -0,081 | 0,201 | -0,474 | 0,318 | -121,21 | 0,011 | 0,228 | -0,442 | 0,458 | -147,64 | -26,43 | 6,72 | -3,93 |  |
|  | **Linear Comp.** | 0,086 | 0,022 | 0,042 | 0,127 |  | 0,024 | 0,008 | 0,009 | 0,04 |  |  |  |  |  |
|  | **Quad. Comp** | -0,001 | 0,001 | -0,002 | 0,001 |  | NA | NA | NA | NA | NA |  |  |  |  |
| **Butterfly Reverse** | **Intercept** | -0,081 | 0,201 | -0,474 | 0,318 | -121,21 | 0,011 | 0,228 | -0,442 | 0,458 | -147,64 | -26,43 | 6,72 | -3,93 |  |
|  | **Linear Comp.** | 0,086 | 0,022 | 0,042 | 0,127 |  | 0,024 | 0,008 | 0,009 | 0,04 |  |  |  |  |  |
|  | **Quad. Comp** | -0,001 | 0,001 | -0,002 | 0,001 |  | NA | NA | NA | NA | NA |  |  |  |  |
| **External Rotation** | **Intercept** | 0,236 | 0,198 | -0,161 | 0,62 | -435,61 | 0,524 | 0,215 | 0,096 | 0,934 | -556,53 | -120,92 | 22,11 | -5,47 |  |
|  | **Linear Comp.** | 0,064 | 0,015 | 0,035 | 0,095 |  | 0,029 | 0,007 | 0,016 | 0,043 |  |  |  |  |  |
|  | **Quad. Comp** | -0,001 | 0,001 | -0,002 | 0 |  | NA | NA | NA | NA | NA |  |  |  |  |
| **Internal Rotation** | **Intercept** | 0,077 | 0,223 | -0,355 | 0,517 | -635,02 | 0,514 | 0,256 | 0,006 | 1,017 | -702,05 | -67,03 | 14,42 | -4,65 |  |
|  | **Linear Comp.** | 0,086 | 0,02 | 0,046 | 0,124 |  | 0,027 | 0,008 | 0,011 | 0,042 |  |  |  |  |  |
|  | **Quad. Comp** | -0,001 | 0,001 | -0,002 | 0 |  | NA | NA | NA | NA | NA |  |  |  |  |
| **Knee Extension** | **Intercept** | 0,101 | 0,186 | -0,273 | 0,449 | -459,36 | 0,147 | 0,22 | -0,297 | 0,572 | -542,88 | -83,52 | 15,65 | -5,34 |  |
|  | **Linear Comp.** | 0,086 | 0,016 | 0,054 | 0,118 |  | 0,033 | 0,008 | 0,018 | 0,047 |  |  |  |  |  |
|  | **Quad. Comp** | -0,001 | 0,001 | -0,002 | 0,001 |  | NA | NA | NA | NA | NA |  |  |  |  |
| **Knee Flexion** | **Intercept** | 0,058 | 0,17 | -0,274 | 0,398 | -422,96 | 0,211 | 0,181 | -0,141 | 0,57 | -488,16 | -65,2 | 13 | -5,02 |  |
|  | **Linear Comp.** | 0,085 | 0,017 | 0,052 | 0,117 |  | 0,031 | 0,007 | 0,017 | 0,045 |  |  |  |  |  |
|  | **Quad. Comp** | -0,001 | 0,001 | -0,002 | 0 |  | NA | NA | NA | NA | NA |  |  |  |  |
| **Latissimus Pull** | **Intercept** | 0,216 | 0,175 | -0,138 | 0,558 | -347,19 | 0,447 | 0,174 | 0,107 | 0,797 | -446,25 | -99,06 | 17,6 | -5,63 |  |
|  | **Linear Comp.** | 0,074 | 0,014 | 0,045 | 0,101 |  | 0,034 | 0,007 | 0,021 | 0,046 |  |  |  |  |  |
|  | **Quad. Comp** | -0,001 | 0,001 | -0,003 | 0 |  | NA | NA | NA | NA | NA |  |  |  |  |
| **Leg Press** | **Intercept** | -0,008 | 0,179 | -0,357 | 0,336 | -65,8 | 0,079 | 0,186 | -0,287 | 0,466 | -103,82 | -38,02 | 9,84 | -3,86 |  |
|  | **Linear Comp.** | 0,052 | 0,011 | 0,032 | 0,072 |  | 0,032 | 0,006 | 0,02 | 0,043 |  |  |  |  |  |
|  | **Quad. Comp** | -0,001 | 0 | -0,001 | 0 |  | NA | NA | NA | NA | NA |  |  |  |  |
| **Retroversion** | **Intercept** | 0,118 | 0,207 | -0,299 | 0,525 | -100,67 | 0,161 | 0,216 | -0,263 | 0,587 | -120,59 | -19,93 | 7,93 | -2,51 |  |
|  | **Linear Comp.** | 0,07 | 0,022 | 0,027 | 0,113 |  | 0,024 | 0,008 | 0,008 | 0,039 |  |  |  |  |  |
|  | **Quad. Comp** | -0,001 | 0,001 | -0,002 | 0 |  | NA | NA | NA | NA | NA |  |  |  |  |
| **Rowing** | **Intercept** | 0,158 | 0,213 | -0,262 | 0,58 | -567,49 | 0,571 | 0,267 | 0,039 | 1,094 | -655,54 | -88,05 | 13,58 | -6,48 |  |
|  | **Linear Comp.** | 0,101 | 0,017 | 0,067 | 0,134 |  | 0,033 | 0,008 | 0,018 | 0,048 |  |  |  |  |  |
|  | **Quad. Comp** | -0,001 | 0,001 | -0,002 | 0 |  | NA | NA | NA | NA |  |  |  |  |  |

^a^ elpd loo: leave one out cross validation estimate of the expected log pointwise predictive density

^b^ elpd diff: difference between elpd loo of the quadratic and the linear model. Negative values display favor for the quadratic model

^c^ Standard error of elpd difference

^d^ Stand. Difference: elpd Difference standardized by Standard error of elpd difference
