# Supplementary material for: Variability in resistance training trajectories of breast cancer patients undergoing therapy
Source: Support Care Cancer. 2024 Dec 10;33(1):12. doi: 10.1007/s00520-024-09001-4 (PMC11631991; doi:10.1007/s00520-024-09001-4)
Supplement: Supplementary file 6 — Supplementary file6 (DOCX 26 KB) [file 520_2024_9001_MOESM6_ESM.docx]

**Variability in resistance training trajectories of breast cancer patients undergoing therapy**

Maximilian Koeppel^1,2^, Karen Steindorf^3^, Martina E. Schmidt^3^, Friederike Rosenberger^2^, Joachim Wiskemann^2^

^1^Institute of Sports and Sport Science, Heidelberg University, Heidelberg, Germany

^2^Working Group Exercise Oncology, Department of Medical Oncology, National Cent

er for Tumor Diseases Heidelberg (NCT Heidelberg) and Heidelberg University Hospital, Heidelberg Germany ^3^Division of Physical Activity, Prevention and Cancer, German Cancer Research Center (DKFZ) and National Center for Tumor Diseases (NCT) Heidelberg, Heidelberg, Germany

|  | Posterior Mean | Posterior SD | -95% UI | +95% UI |
| --- | --- | --- | --- | --- |
| **Population Level Effect, Constants** | | | | |
| **Intercept** | 0,020 | 0,102 | -0,200 | 0,204 |
| **Linear Component** | 0,090 | 0,015 | 0,056 | 0,116 |
| **Quadratic Component** | -0,002 | 0,001 | -0,003 | -0,001 |
| **Variation between Exercise** | | | | |
| **Intercept** | 0,183 | 0,115 | 0,012 | 0,443 |
| **Linear Component** | 0,036 | 0,014 | 0,014 | 0,013 |
| **Quadratic Component** | 0,001 | 0,001 | <0,001 | 0,003 |
| **Variation between Individuals** | | | | |
| **Intercept** | 0,990 | 0,045 | 0,907 | 1,085 |
| **Linear Component** | 0,150 | 0,008 | 0,135 | 0,165 |
| **Quadratic Component** | 0,007 | <0,001 | 0,006 | 0,008 |

*Supplementary Information 6 - Adherence Data*

*Adherence to 12 (50%) of total number of sessions (n= 38)*

*Adherence to 16 (75%) of total number of sessions (n= 27)*

|  | Posterior Mean | Posterior SD | -95% UI | +95% UI |
| --- | --- | --- | --- | --- |
| **Population Level Effect, Constants** | | | | |
| **Intercept** | -0,034 | 0,118 | -0,296 | 0,179 |
| **Linear Component** | 0,091 | 0,015 | 0,058 | 0,117 |
| **Quadratic Component** | -0,002 | <0,001 | -0,003 | -0,001 |
| **Variation between Exercise** | | | | |
| **Intercept** | 0,222 | 0,127 | 0,022 | 0,511 |
| **Linear Component** | 0,036 | 0,014 | 0,014 | 0,069 |
| **Quadratic Component** | 0,001 | 0,001 | <0,001 | 0,002 |
| **Variation between Individuals** | | | | |
| **Intercept** | 0,980 | 0,052 | 0,883 | 1,085 |
| **Linear Component** | 0,137 | 0,008 | 0,122 | 0,154 |
| **Quadratic Component** | 0,006 | <0,001 | 0,005 | 0,007 |

***Conclusion***

Including only patients with high adherence did not have a significant impact on the parameter estimates of the model.
