# Supplementary material for: Variability in resistance training trajectories of breast cancer patients undergoing therapy
Source: Support Care Cancer. 2024 Dec 10;33(1):12. doi: 10.1007/s00520-024-09001-4 (PMC11631991; doi:10.1007/s00520-024-09001-4)
Supplement: Supplementary file 7 — Supplementary file7 (DOCX 19 KB) [file 520_2024_9001_MOESM7_ESM.docx]

**Variability in resistance training trajectories of breast cancer patients undergoing therapy**

Maximilian Koeppel^1,2^, Karen Steindorf^3^, Martina E. Schmidt^3^, Friederike Rosenberger^2^, Joachim Wiskemann^2^

^1^Institute of Sports and Sport Science, Heidelberg University, Heidelberg, Germany

^2^Working Group Exercise Oncology, Department of Medical Oncology, National Center for Tumor Diseases Heidelberg (NCT Heidelberg) and Heidelberg University Hospital, Heidelberg Germany

^3^Division of Physical Activity, Prevention and Cancer, German Cancer Research Center (DKFZ) and National Center for Tumor Diseases (NCT) Heidelberg, Heidelberg, Germany

*Supplementary Information 7 - Frequency of positive posterior means per exercise*

*Table S7.1. Absolute frequency and proportion of positive posterior means of the linear component of patients with valid data on a particular exercise*

| **Exercise** | **frequency** | **Proportion in %** | **Total number of patients** |
| --- | --- | --- | --- |
| Anteversion | 31 | 93,9 | 33 |
| Butterfly | 63 | 95,5 | 66 |
| Butterfly Reverse | 28 | 82,4 | 34 |
| External Rotation | 46 | 68,7 | 67 |
| Internal Rotation | 51 | 76,1 | 67 |
| Knee Extension | 46 | 66,7 | 69 |
| Knee Flexion | 48 | 69,6 | 69 |
| Lat Pull Down | 54 | 78,3 | 69 |
| Leg Press | 61 | 88,4 | 69 |
| Retroversion | 30 | 85,7 | 35 |
| Rowing | 51 | 73,9 | 69 |
