# Supplementary material for: Variability in resistance training trajectories of breast cancer patients undergoing therapy
Source: Support Care Cancer. 2024 Dec 10;33(1):12. doi: 10.1007/s00520-024-09001-4 (PMC11631991; doi:10.1007/s00520-024-09001-4)
Supplement: Supplementary file 8 — Supplementary file8 (PPTX 3055 KB) [file 520_2024_9001_MOESM8_ESM.pptx]

## Slide 1
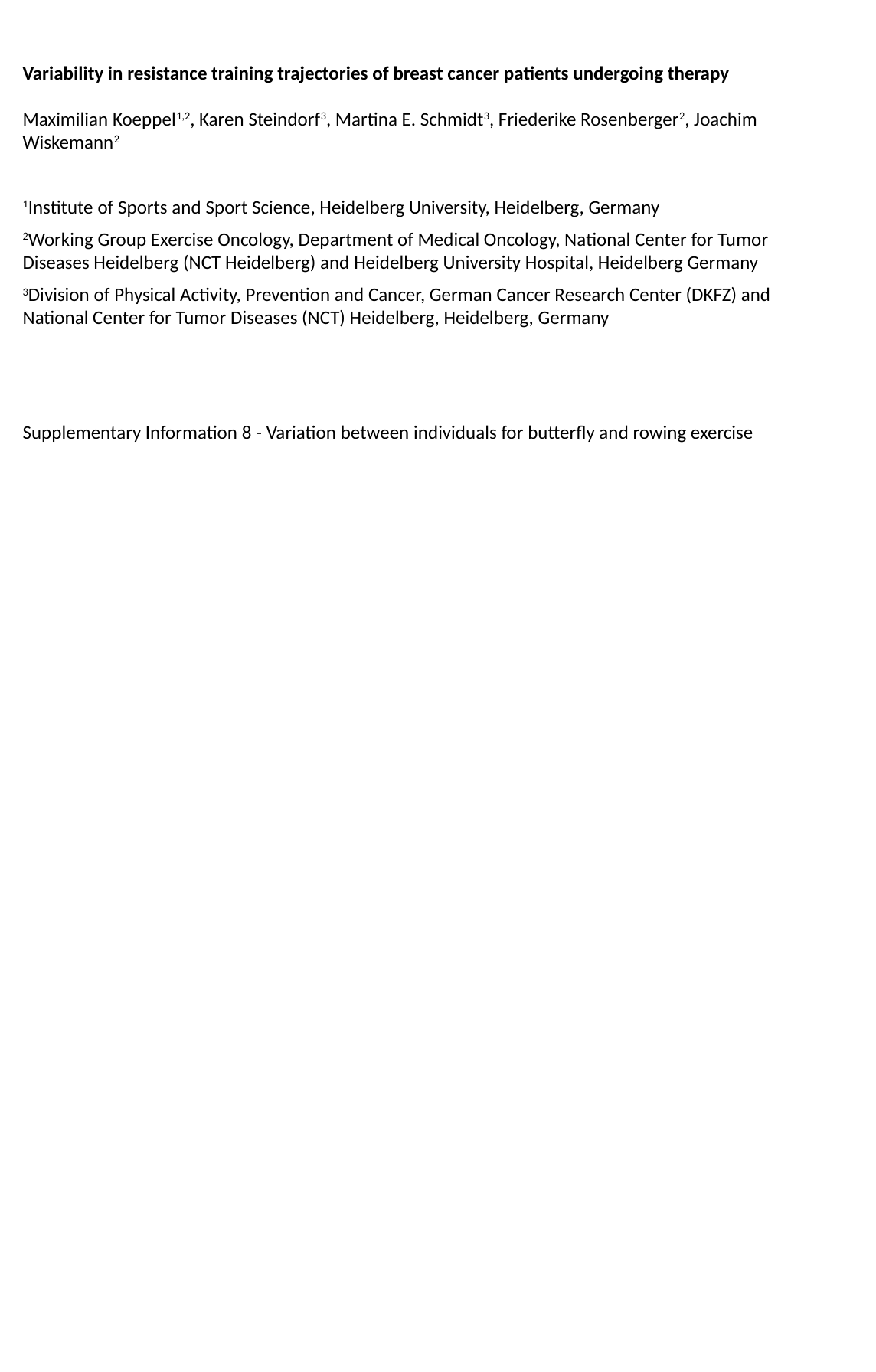

Variability in resistance training trajectories of breast cancer patients undergoing therapy
Maximilian Koeppel1,2, Karen Steindorf3, Martina E. Schmidt3, Friederike Rosenberger2, Joachim Wiskemann2
1Institute of Sports and Sport Science, Heidelberg University, Heidelberg, Germany
2Working Group Exercise Oncology, Department of Medical Oncology, National Center for Tumor Diseases Heidelberg (NCT Heidelberg) and Heidelberg University Hospital, Heidelberg Germany
3Division of Physical Activity, Prevention and Cancer, German Cancer Research Center (DKFZ) and National Center for Tumor Diseases (NCT) Heidelberg, Heidelberg, Germany
Supplementary Information 8 - Variation between individuals for butterfly and rowing exercise

## Slide 2
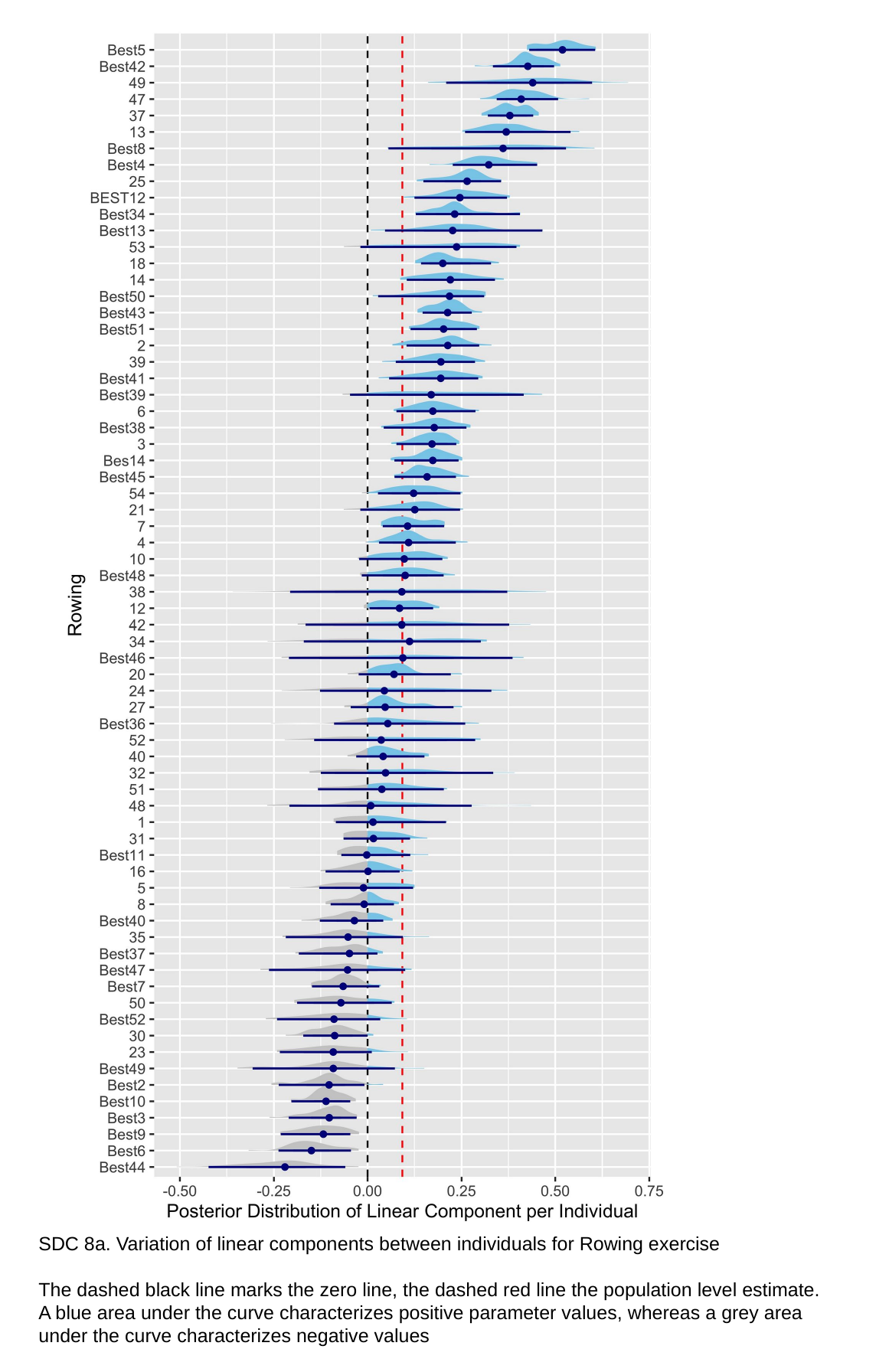

SDC 8a. Variation of linear components between individuals for Rowing exercise
The dashed black line marks the zero line, the dashed red line the population level estimate. A blue area under the curve characterizes positive parameter values, whereas a grey area under the curve characterizes negative values

## Slide 3
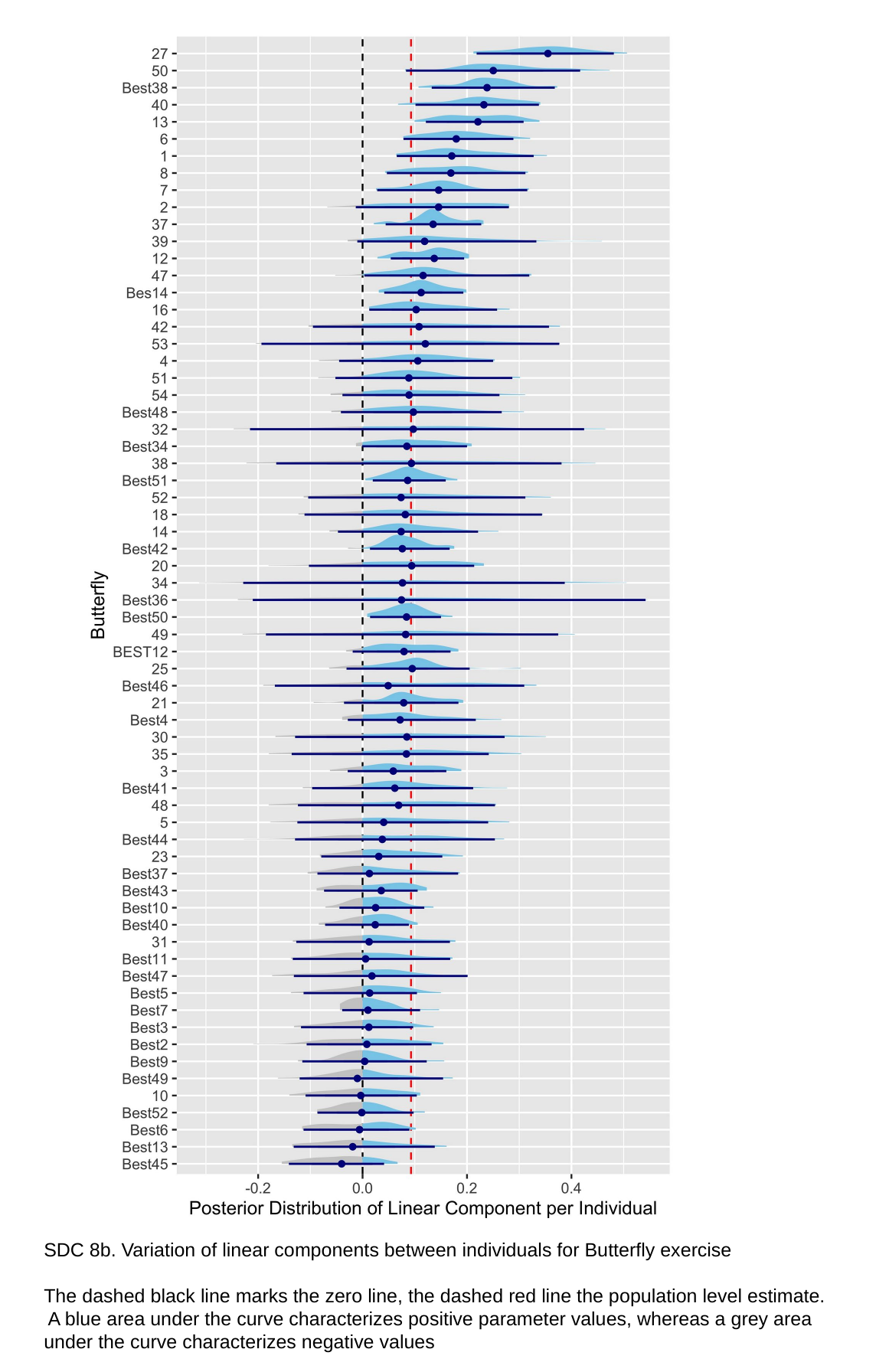

SDC 8b. Variation of linear components between individuals for Butterfly exercise
The dashed black line marks the zero line, the dashed red line the population level estimate. A blue area under the curve characterizes positive parameter values, whereas a grey area under the curve characterizes negative values
