# Supplementary material for: Variability in resistance training trajectories of breast cancer patients undergoing therapy
Source: Support Care Cancer. 2024 Dec 10;33(1):12. doi: 10.1007/s00520-024-09001-4 (PMC11631991; doi:10.1007/s00520-024-09001-4)
Supplement: Supplementary file 9 — Supplementary file9 (DOCX 12 KB) [file 520_2024_9001_MOESM9_ESM.docx]

**Variability in resistance training trajectories of breast cancer patients undergoing therapy**

Maximilian Koeppel^1,2^, Karen Steindorf^3^, Martina E. Schmidt^3^, Friederike Rosenberger^2^, Joachim Wiskemann^2^

^1^Institute of Sports and Sport Science, Heidelberg University, Heidelberg, Germany

^2^Working Group Exercise Oncology, Department of Medical Oncology, National Center for Tumor Diseases Heidelberg (NCT Heidelberg) and Heidelberg University Hospital, Heidelberg Germany

^3^Division of Physical Activity, Prevention and Cancer, German Cancer Research Center (DKFZ) and National Center for Tumor Diseases (NCT) Heidelberg, Heidelberg, Germany

*Supplementary Information 9 - CERT Checklist*

*Tab. 9.1. CERT Checklist*

| **Item** | **Description** | **Study Information** |
| --- | --- | --- |
| **1** | Detailed description of the type of exercise equipment | A machine based exercise program with a core set of exercises: leg press (linear machine), leg extension (rotatory machine), leg curl (rotatory machine), shoulder internal (rope pull) and external rotation (rope pull), seated row (linear machine), latissimus pull down (linear machine) and butterfly (rope pull)  Warmup was conducted on a stationary bike or by rowing for 5 minutes  In addition to the core set of exercises, patients in the BEATE-Study also conducted butterfly reverse (Rope pull) and anteversion (rope pull) or retroversion (rope pull) of the shoulder alternating every other TS each training session. |
| **2** | Detailed description of the qualifications, expertise and/or training | All sessions were supervised by two physiotherapists with experience in working with oncologic patients. One of the therapist is also a trained exercise physiologist on a master-degree level. |
| **3** | Describe whether exercises are performed individually or in a group | The two physiotherapists supervised groups of patients. However, since patients were allowed to join the training freely the number of participants varied from day to day. |
| **4** | Describe whether exercises are supervised or unsupervised; how they are delivered | Sessions were conducted in the facility’s fitness centre and supervised by two trained physical therapists experienced in working with cancer patients. |
| **5** | Detailed description of how adherence to exercise is measured and reported | Each patient protocoled their attendance at the beginning of each session. The attendance list was checked daily by the therapists. Regarding training adherence, each patient had a training log and documented repetitions, load and sets for each exercise. The training log was checked by the therapists several times during the training session. |
| **6** | Detailed description of motivation strategies | No motivational strategies were applied. |
| **7a** | Detailed description of the decision rule(s) for determining exercise prorgression | The load was increased by at least 5% if a patient was able to complete three sets of twelve repetitions of an exercise in three consecutive training sessions. |
| **7b** | Detailed description of how the exercise program was progressed | The patients started out with a particular load and should perform 1-3 sets à 12 repetitions. When they were able to conduct 12 repetition in each of the sets, the training load was increased by … |
| **8** | Detailed description of of each exercise to enable replication | A machine based exercise program with a core set of exercises: leg press (linear machine), leg extension (rotatory machine), leg curl (rotatory machine), shoulder internal (rope pull) and external rotation (rope pull), seated row (linear machine), latissimus pull down (linear machine) and butterfly (rope pull).  In addition to the core set of exercises, patients in the BEATE-Study also conducted butterfly reverse (Rope pull) and anteversion (rope pull) or retroversion (rope pull) of the shoulder alternating every other TS each training session. |
| **9** | Detailed description of any home program component | No home program was conducted. |
| **10** | Describe whether there are any non-exercise components | The patients exclusively conducted a supervised exercise. |
| **11** | Describe the type and number of adverse events that occur  during exercise | Potential adverse effects (e.g. lymphedema, pain, muscle soreness, nausea, dyspnea and tachycardia) were recorded by patients during each training session using standardized questionnaires. Serious adverse events were recorded by the therapists. No injuries or severe adverse events were reported by either the patients nor the therapists. The frequency of self reported adverse events (e.g. lymphedema, pain, muscle soreness, nausea, dyspnea and tachycardia) did not differ between groups (p>.05). |
| **12** | Describe the setting in which the exercises are performed | The sessions were conducted in the facility’s gym, under supervision of two experienced physiotherapists. |
| **13** | Detailed description of the exercise intervention | Over the course of twelve weeks, patients conducted a supervised machine based progressive resistance training program twice a week. The progressive resistance training program consisted of 8 exercises in the BEST study and 11 exercises (only 10 exercises were conducted in a single training session) in the BEATE Study. Patients were asked to conduct 3 sets à 12 repetitions of each exercise at 60-80% of their hypothetical 1RM, estimated with the Brzycki-Formula. Between sets, patients were asked to include a resting break of 1 minute, thus, the total duration for a complete TS accumulated to approximately 60 minutes. The training schedule followed a progressive approach, in which the applied load was increased by at least 5% if the prescribed load was successfully achieved for 3 sets of 12 repetitions in three consecutive TS. |
| **14a** | Describe whether the exercises are generic (one size fits all) or  tailored | The exercise program was standardized for all participants. |
| **14b** | Detailed description of how exercises are tailored to the individual | Only minor adaptations were conducted, if patients reported pain. |
| **15** | Describe the decision rule for determining the starting level | The starting weight matched 60% of the patients hypothetical RM, estimated via Brzycki-Formula and based on the load and repetition noted in the two familiarization sessions at the beginning of the intervention. |
| **16** | Describe how adherence or fidelity is assessed/measured | The physiotherapists were involved in the development of the training schedule. Half a day trained… Each week therapists gave a report of the training sessions and discussed any issues that occurred during the sessions in a large team of exercise professionals. |
| **16b** | Describe the extent to which the intervention was delivered as  planned | Patients attended a median number of 20 TS (IQR: 14-23) for a maximum of 24 sessions. |
