# Supplementary material for: Variability in resistance training trajectories of breast cancer patients undergoing therapy
Source: Support Care Cancer. 2024 Dec 10;33(1):12. doi: 10.1007/s00520-024-09001-4 (PMC11631991; doi:10.1007/s00520-024-09001-4)
Supplement: Supplementary file 10 — Supplementary file10 (DOCX 10 KB) [file 520_2024_9001_MOESM10_ESM.docx]

**Variability in resistance training trajectories of breast cancer patients undergoing therapy**

Maximilian Koeppel^1,2^, Karen Steindorf^3^, Martina E. Schmidt^3^, Friederike Rosenberger^2^, Joachim Wiskemann^2^

^1^Institute of Sports and Sport Science, Heidelberg University, Heidelberg, Germany

^2^Working Group Exercise Oncology, Department of Medical Oncology, National Center for Tumor Diseases Heidelberg (NCT Heidelberg) and Heidelberg University Hospital, Heidelberg Germany

^3^Division of Physical Activity, Prevention and Cancer, German Cancer Research Center (DKFZ) and National Center for Tumor Diseases (NCT) Heidelberg, Heidelberg, Germany

*Supplementary Information 10 - Raw Baseline Volume-Load for all Exercises*

*Table S10.1. Raw Baseline Volume-Load for all Exercises*

| **Exercise** | **Leg Press** | **Knee Extension** | **Knee Flexion** | **Rowing** | **Latissimus Pull** | **Internal Rotation** |
| --- | --- | --- | --- | --- | --- | --- |
| **Mean** | 1537 | 535 | 270 | 481 | 564 | 173 |
| **SD** | 423 | 216 | 107 | 124 | 168 | 41 |
| **25th Percentile** | 1332 | 317 | 198 | 360 | 421 | 144 |
| **Median** | 1620 | 634 | 198 | 480 | 540 | 162 |
| **75th Percentile** | 1908 | 634 | 396 | 540 | 720 | 198 |
|  |  |  |  |  |  |  |
| **Exercise (cont.)** | **External Rotation** | **Butterfly** | **Anteversion** | **Retroversion** | **Butterfly Reverse** |  |
| **Mean** | 136 | 130 | 114 | 160 | 208 |  |
| **SD** | 44 | 86 | 54 | 57 | 56 |  |
| **25th Percentile** | 108 | 36 | 72 | 108 | 180 |  |
| **Median** | 144 | 119 | 108 | 144 | 216 |  |
| **75th Percentile** | 144 | 199 | 144 | 216 | 252 |  |
