# Supplementary material for: Variability in resistance training trajectories of breast cancer patients undergoing therapy
Source: Support Care Cancer. 2024 Dec 10;33(1):12. doi: 10.1007/s00520-024-09001-4 (PMC11631991; doi:10.1007/s00520-024-09001-4)
Supplement: Supplementary file 11 — Supplementary file11 (DOCX 37 KB) [file 520_2024_9001_MOESM11_ESM.docx]

**Variability in resistance training trajectories of breast cancer patients undergoing therapy**

Maximilian Koeppel^1,2^, Karen Steindorf^3^, Martina E. Schmidt^3^, Friederike Rosenberger^2^, Joachim Wiskemann^2^

^1^Institute of Sports and Sport Science, Heidelberg University, Heidelberg, Germany

^2^Working Group Exercise Oncology, Department of Medical Oncology, National Center for Tumor Diseases Heidelberg (NCT Heidelberg) and Heidelberg University Hospital, Heidelberg Germany

^3^Division of Physical Activity, Prevention and Cancer, German Cancer Research Center (DKFZ) and National Center for Tumor Diseases (NCT) Heidelberg, Heidelberg, Germany

**Supplementary Information 11 - Progression Formula**

In our paper we applied a quadratic model to the exercise training data of 69 breast cancer patients undergoing adjuvant cancer treatment. Our rationale for applying a quadratic model, as outlined in the main document was the well known phenomenon, that resistance training gains are larger in the beginning of an intervention, due to neuro-muscular adaptations such as inter muscular coordination, than in the later course of the intervention (Sale, . Steele). However, we are aware, that the quadratic model does not mimic the true training progression trajectory of the patients. For instance, as it is pointed out in the discussion of the main document, the function will show a negative slope after reaching its vertex, which of course is not the case in the patients’ training trajectory. Therefore one can think of other, potentially more appropriate statistical models. The downside of these models is, however, that they are much more difficult to understand, to construct and to fit to the data. Furthermore, the practical value of these models, remains questionable. Therefore we would refer to the famous aphorism, often assigned to statistician George E. P. Box: „All models are wrong, but some are useful“. And we believe the quadratic model can be of great use in exercise management, especially in planning progression and periodization schemes.

Our main analysis resulted in the following function (For the sake of simplicity, we are only looking at the posterior mean values and ignore their uncertainty)

*y = 0.093*t - 0.002*t^2 (1)*

Since we z-standardized the dependent variable, both factors 0.093 and 0.002 can be interpreted as proportions of the standard deviation of the initial load. Regarding the baseline data (as provided in Supplemental Information SI 10), the linear component equals 3-5% of the initial load. Considering that most resistance training machines will not allow for a gradual adjustment of the trainings load, the progression will only take place after a couple of sessions. Therefore we would argue, a progression in load of 5%, as often done by convention, is justified. The quadratic component, which is roughly 1/50th of the linear component (0.093/0.002 = 46.5), becomes 0.1% (0.05/50 = .001) of the initial load. Therefore the function can be translated into to determine what load should be applied when progressing, based on the initial load L0:

L_Prog_ = L_0_ * 0.05 * t - L_0_ * 0.001 * t^2^ (2)

Formula (2) describes the increase in training load at the progression t, if one is interested in the absolute load L_Abs_ at progression t, the initial load need so be added.

*L_Abs_ = L_0_ + L_0_ * 0.05 * t - L_0_ * 0.001 * t^2^ (3)*

Alternatively we can construct a modified exponential growth model, that might be more intuitive then the quadratic model, since it takes the initial relative progression and the correction factor in context of the initial relative progression into account. The formula for the absolute load at progression t can be calculated as:

*L_Abs_ = L_0_* (1 + p * (1-q)^t-1^ )^t^ (4)*

As above L(t) is the load after progression t, L0 is he initial Load, q is the initial progression factor (e.g. 5%) and q is the correction factor by which the initial progression factor is adjusted.

Both formula (3) and (4) show similar curves for the time span of interest.


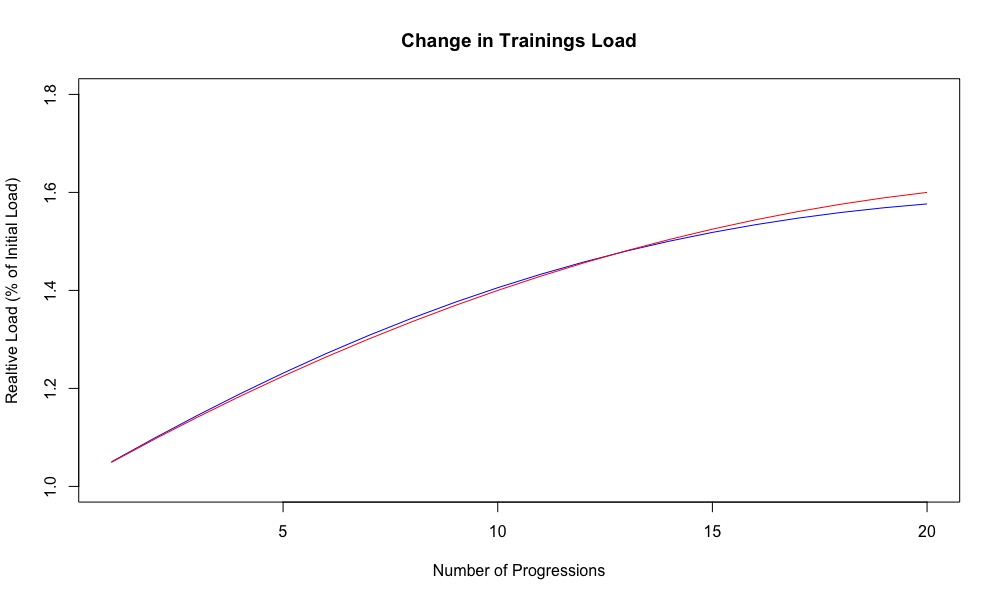


Fig. 11.1 Progression in trainings load. The red curve equals equation (3), the blue curve equation (4)

The R-code for formula (3) and (4) as functions, where:

L0: ist the initial Load

t: Number of progressions

p: Initial progression factor (e.g. 5%)

q: Correction factor (e.g. 4%)

Training_load_quadratic <- function(L0, t, p) {

return(L0 + L0 * p * t - L0 * p/50 * t^2)

}

Training_load_exponential <- function(B0, t, p, q) {

return(L0 + L0 * p * t - B0* q * t^2)

}

Training_load_exponential <- function(B0, t, p, q) {

return(L0 * (1 + p * (1 - q) ^ (t - 1)) ^ t)

}
